# Can large language models accurately compute descriptive statistics from structured datasets? A comparative evaluation of ChatGPT and Claude

**DOI:** 10.1016/j.ahjo.2026.100877

**Published:** 2026-08-27

**Authors:** Paul Sebo, Ting Wang

**Affiliations:** a University Institute for primary care (IuMFE), University of Geneva, Geneva, Switzerland; b School of Library and Information Management, Emporia State University, Emporia, KS, USA

**Keywords:** AI, Artificial intelligence, ChatGPT, Claude, Descriptive statistic, Large language model, LLM, Research, Statistical analysis

## Abstract

**Background:**

Large language models (LLMs) are increasingly used to support statistical analyses in biomedical research. However, their ability to accurately and reproducibly compute descriptive statistics directly from datasets has received limited evaluation.

**Objective:**

To compare the accuracy, within-modality repeatability, and between-modality consistency of ChatGPT and Claude in generating descriptive statistics from structured datasets provided through different input modalities.

**Methods:**

Two publicly available Stata datasets were evaluated: ‘auto.dta’ (74 observations, 12 variables) and ‘citytemp.dta’ (956 observations, 6 variables). Original variable names were replaced with generic labels. Each dataset was analyzed using three input modalities (copy-paste, Word, and Excel), with two independent repetitions per modality. Stata served as the reference standard. Accuracy was assessed for counts of missing and non-missing observations, minima, maxima, means, standard deviations, medians, quartiles, frequencies, and percentages.

**Results:**

ChatGPT and Claude produced identical results across all analyses. For each model, 624 categories of descriptive statistics were evaluated. Exact agreement with the Stata reference standard was observed for 576 of 624 categories (92.3%). The only deviations involved first and third quartiles (Q1–Q3) for eight variables. Post hoc analyses demonstrated that these differences were entirely attributable to the use of a different, but mathematically valid, quartile definition based on linear interpolation rather than computational errors. Within-modality repeatability and between-modality consistency were complete across the evaluated analyses.

**Conclusions:**

ChatGPT and Claude demonstrated excellent accuracy and consistent results across repeated analyses and input modalities for the datasets and descriptive statistics evaluated. After accounting for differences in quartile definitions, no calculation errors were identified across the 624 evaluated categories per model.

## Highlights

• ChatGPT and Claude showed high accuracy for descriptive statistics.
• Outputs were identical across input modalities and repeated analyses.
• Deviations were limited to Q1–Q3 estimates for eight variables.
• Quartile deviations reflected valid methodological differences.
• No calculation errors were found after accounting for quartile definitions.

## 1 Introduction

Artificial intelligence (AI) is increasingly integrated into biomedical research and clinical practice. Large language models (LLMs) such as ChatGPT and Claude provide natural-language interfaces capable of supporting a wide range of tasks, including literature synthesis, scientific writing, clinical decision support, education, and statistical analysis [1], [2], [3], [4], [5], [6], [7], [8], [9], [10], [11], [12], [13]. As these systems become more widely available, researchers are increasingly using them as practical assistants throughout the research workflow. However, the reliability of LLM-generated outputs varies across research tasks. Studies evaluating scientific reference generation, evidence synthesis, and structured clinical reporting have demonstrated task- and model-dependent performance, with persistent concerns regarding accuracy and reliability [14], [15], [16], [17]. These findings highlight the importance of evaluating LLM reliability separately for specific components of biomedical research workflows.

One area of growing interest is the use of LLMs for quantitative and statistical tasks. Accurate statistical analysis is fundamental to medical research, as errors in data handling, calculation, or reporting may lead to incorrect conclusions and reduced reproducibility [18], [19], [20]. Unlike narrative tasks, statistical computations have objectively verifiable answers, making them particularly suitable for benchmarking AI performance.

Several studies have explored the use of LLMs in statistical analysis. LLMs have demonstrated promising performance in selecting appropriate statistical tests, generating statistical software commands, performing data management tasks, and assisting with sample-size estimation [10], [11], [12], [13]. However, existing studies have also identified important limitations, including occasional computational errors, difficulties handling assumptions, inconsistent reasoning, and the risk of producing plausible but incorrect outputs [21], [22]. These concerns are particularly relevant in medical research, where seemingly minor numerical inaccuracies may affect interpretation and reproducibility.

Recently, our group evaluated several leading LLMs for identifying statistical tests and generating corresponding Stata commands and found high levels of accuracy and reproducibility across models [11]. Nevertheless, selecting a statistical test and generating software syntax represent only part of the analytical process. Before any inferential analysis can be performed, researchers routinely calculate descriptive statistics such as means, standard deviations, medians, quartiles, frequencies, and percentages. These calculations are among the most common statistical operations performed in biomedical research and constitute a fundamental prerequisite for data exploration and reporting. Yet, despite their importance, the ability of LLMs to compute descriptive statistics directly from uploaded or pasted datasets has received limited systematic evaluation. Existing studies have primarily focused on a single model, most often ChatGPT. For example, Ruta et al. reported accurate descriptive statistical calculations using ChatGPT but evaluated only a limited set of descriptive measures within a broader assessment of data-management and inferential statistical tasks [13]. Similarly, Meo et al. assessed ChatGPT across a range of biomedical statistical analyses, including descriptive statistics, hypothesis testing, regression modeling, and graphical outputs, but did not specifically investigate accuracy and within-modality repeatability or consistency across different dataset input modalities [23]. Consequently, evidence regarding the reliability of contemporary LLMs for routine descriptive statistical calculations remains limited.

To our knowledge, no study has directly compared leading LLMs for the generation of descriptive statistics from datasets provided through different input modalities. We therefore evaluated the performance of ChatGPT and Claude using two publicly available Stata datasets supplied as copy-pasted tables, Microsoft Word documents, and Microsoft Excel files. Accuracy was assessed against a Stata reference standard, while within-modality repeatability and between-modality consistency were evaluated across repeated analyses and input modalities. We hypothesized that both models would demonstrate high accuracy, repeatability, and consistency across the evaluated input modalities.

## 2 Methods

### 2.1 Study design and data sources

We conducted an experimental study evaluating the ability of two large language models (LLMs), ChatGPT (OpenAI, GPT-5.5) and Claude (Anthropic, Claude Sonnet 4.6), to generate descriptive statistics from structured datasets. Dataset selection, preparation, and data collection were performed between May and June 2026. Both models were accessed through their standard web interfaces rather than through an application programming interface (API), and temperature and other inference parameters were therefore not manually specified or controlled. The prompts instructed the models to provide only the requested numerical results in tables, without explanations or comments (Supplementary Material 1). Consequently, the recorded outputs did not allow us to determine whether calculations were performed solely through model processing, through generated code, or with the assistance of embedded computational tools.

Two publicly available datasets distributed with Stata were selected as test datasets. To evaluate model performance across datasets of different sizes and characteristics, we intentionally selected one dataset containing between 50 and 100 observations and another containing between 500 and 1000 observations. In addition, both datasets included a mixture of continuous and categorical variables.

The first dataset, ‘auto.dta’, contained 74 observations and 12 variables describing automobile characteristics, including vehicle make and model, price, fuel efficiency (miles per gallon), repair record, headroom, trunk space, vehicle weight, vehicle length, turning circle, engine displacement, gear ratio, and domestic versus foreign origin. The second dataset, ‘citytemp.dta’, contained 956 observations and 6 variables describing climatic characteristics of U.S. cities, including census division, census region, heating degree days, cooling degree days, average January temperature, and average July temperature. Both datasets are publicly available example datasets distributed with Stata and can be loaded directly using the commands ‘sysuse auto’ and ‘sysuse citytemp’, respectively. To minimize the possibility that the models would recognize the datasets from prior exposure or training data, all original variable names were replaced with generic labels (V1, V2, V3, etc.) before analysis. The underlying data values remained unchanged.

### 2.2 Reference standard

Stata was used as the reference standard (gold standard) for all analyses. Reference descriptive statistics were generated directly from the original datasets and were considered the correct values against which LLM outputs were compared.

For continuous variables, descriptive statistics were obtained using the Stata command ‘codebook’, which provided the number of non-missing observations, number of missing observations, minimum, maximum, mean, standard deviation, median, and quartiles. For categorical variables, the Stata commands ‘codebook’ and ‘tabulate’ were used to obtain the number of non-missing observations, number of missing observations, category frequencies, and category percentages. The reference descriptive statistics are presented in Supplementary Material 2, while the corresponding Stata output is provided in Supplementary Material 5. All reference analyses were performed using Stata version 15 (StataCorp, College Station, TX, USA).

### 2.3 Experimental conditions

Each dataset was evaluated using three input modalities: direct copy-paste of the dataset into the chat interface, an attached Microsoft Excel file, and an attached Microsoft Word file containing the dataset.

Six standardized prompts were developed and applied identically across both LLMs. Three prompts were used for the ‘auto.dta’ dataset (copy-paste input, Excel file input, and Word file input), and three prompts were used for the ‘citytemp.dta’ dataset (copy-paste input, Excel file input, and Word file input). The full prompts are provided in Supplementary Material 1.

For the ‘auto.dta’ dataset, variable V1 (‘make’) was intentionally excluded from analysis, because it corresponded to the vehicle name and therefore represented a unique text identifier rather than a categorical or continuous variable suitable for descriptive statistical analysis. Variables V4, V5, and V12 were specified as categorical variables, whereas variables V2, V3, V6, V7, V8, V9, V10, and V11 were specified as continuous variables.

For the ‘citytemp.dta’ dataset, variables V1 and V2 were specified as categorical variables, whereas variables V3 through V6 were specified as continuous variables.

For categorical variables, the models were instructed to report the number of non-missing observations, number of missing observations, category frequencies, and category percentages. For continuous variables, the models were instructed to report the number of non-missing observations, number of missing observations, minimum, maximum, mean, standard deviation, median, and first and third quartiles (Q1–Q3).

### 2.4 Study procedures

For both LLMs, each dataset was analyzed using all three input modalities (copy-paste, Excel, and Word). To assess within-modality repeatability, each analysis was repeated twice in independent chat sessions using the same prompt and the same dataset.

Consequently, each LLM performed six analyses per dataset: two copy-paste analyses, two Excel-file analyses, and two Word-file analyses. Thus, each LLM completed 12 analyses overall across the two datasets.

### 2.5 Outcome assessment

The primary outcome was the accuracy of descriptive statistics generated by each LLM compared with the Stata reference values.

The categories of descriptive statistics evaluated depended on the variable type. For continuous variables, the models were asked to report seven categories of descriptive statistics: (1) number of non-missing observations, (2) number of missing observations, (3) minimum and maximum values, (4) mean, (5) standard deviation, (6) median, and (7) first and third quartiles (Q1–Q3). For categorical variables, the models were asked to report four categories of descriptive statistics: (1) number of non-missing observations, (2) number of missing observations, (3) category frequencies, and (4) category percentages.

To standardize comparisons across models, all decimal values were recorded with two digits after the decimal point. If the absolute difference between an LLM-generated value and the corresponding Stata reference value was less than 0.01, the result was considered correct and attributed to rounding differences.

Each category of descriptive statistics was evaluated independently. For categories containing multiple values (e.g., Min–Max, Q1–Q3, category frequencies, or category percentages), all values within that category had to be correct for the category to be classified as correct. If at least one value within a category was incorrect, the entire category was classified as incorrect.

Each analysis of ‘auto.dta’ included 68 evaluable categories, whereas each analysis of ‘citytemp.dta’ included 36 evaluable categories. Because each dataset was evaluated six times per model (two copy-paste analyses, two Excel-file analyses, and two Word-file analyses), a total of 408 evaluable categories were assessed for ‘auto.dta’ and 216 evaluable categories were assessed for ‘citytemp.dta’. Consequently, a total of 624 evaluable categories were assessed for each LLM.

### 2.6 Post hoc analysis of quartile definitions

Following completion of the primary analyses, a post hoc investigation was conducted to explore the source of the observed Q1–Q3 discrepancies. Two additional sets of prompts were developed and applied to ChatGPT and Claude. In the first set, the models were explicitly instructed to calculate quartiles using the same percentile definition as Stata's ‘codebook’ and ‘summarize, detail’ commands. Specifically, for a dataset containing n non-missing observations and a percentile p, the position was calculated as P = n × p. If P was an integer, the percentile was defined as the average of the observations at ranks P and P + 1 in the sorted data. If P was not an integer, the percentile was defined as the observation at the first rank strictly greater than P.

In the second set, the models were explicitly instructed to calculate quartiles using a linear interpolation approach. Specifically, for a dataset containing n non-missing observations and percentile p, the percentile position was calculated as h = 1 + (n − 1) × p. If h was an integer, the percentile was defined as the value at rank h. If h was not an integer, let k denote the integer part of h and d = h − k; the percentile was then calculated as x(k) + d × [x(k + 1) − x(k)], where x(k) and x(k + 1) represent the sorted observations at ranks k and k + 1, respectively. This procedure corresponded exactly to the quartile values generated by ChatGPT and Claude in the primary analyses.

The objective of these analyses was to determine whether the discrepancies observed in the primary analyses reflected computational errors or the use of an alternative, but valid, quartile definition. The complete prompts used for these post hoc analyses are provided in Supplementary Material 1.

### 2.7 Statistical analysis

For each requested category of descriptive statistics, results generated by the LLMs were classified as correct or incorrect according to the predefined agreement criterion.

Overall accuracy was calculated as the proportion of correctly reported categories among all evaluable categories. Within-modality repeatability was assessed descriptively by comparing outputs from the two independent repetitions performed with the same input modality. Between-modality consistency was assessed by comparing outputs obtained from copy-paste, Word, and Excel inputs. Because ChatGPT and Claude produced identical results across all analyses, no formal statistical comparisons between models were performed. All analyses were performed using Stata version 15 (StataCorp, College Station, TX, USA).

## 3 Results

Table 1 provides variable-level accuracy data, including the number and proportion of deviating categories for each variable, and summarizes consistency across the six analyses performed for each dataset. Supplementary Material 2 presents the Stata reference descriptive statistics, while Supplementary Materials 3 and 4 provide the complete outputs generated by ChatGPT and Claude, respectively. Because both models produced identical results across all datasets, input modalities, and repeated runs, Supplementary Materials 3 and 4 are identical.

**Table 1:** Accuracy and consistency of ChatGPT and Claude relative to the Stata reference standard across six analyses per dataset.

Dataset	Variable	Categories evaluated, n	Deviating categories, n (%)	Consistent across all 6 analyses per dataset	Description of deviation
auto.dta	V1 (make)	N/A	N/A	N/A	N/A
auto.dta	V2 (price)	7	1 (14.3)	Yes	Q1-Q3
auto.dta	V3 (mpg)	7	1 (14.3)	Yes	Q1-Q3
auto.dta	V4 (rep78)	4	0	Yes	No deviation
auto.dta	V5 (headroom)	4	0	Yes	No deviation
auto.dta	V6 (trunk)	7	1 (14.3)	Yes	Q1-Q3
auto.dta	V7 (weight)	7	1 (14.3)	Yes	Q1-Q3
auto.dta	V8 (length)	7	1 (14.3)	Yes	Q1-Q3
auto.dta	V9 (turn)	7	0	Yes	No deviation
auto.dta	V10 (displacement)	7	1 (14.3)	Yes	Q1-Q3
auto.dta	V11 (gear_ratio)	7	1 (14.3)	Yes	Q1-Q3
auto.dta	V12 (foreign)	4	0	Yes	No deviation
citytemp.dta	V1 (division)	4	0	Yes	No deviation
citytemp.dta	V2 (region)	4	0	Yes	No deviation
citytemp.dta	V3 (heatdd)	7	0	Yes	No deviation
citytemp.dta	V4 (cooldd)	7	0	Yes	No deviation
citytemp.dta	V5 (tempjan)	7	1 (14.3)	Yes	Q1–Q3
citytemp.dta	V6 (tempjuly)	7	0	Yes	No deviation
Total (distinct categories across both datasets)		104	8 (7.7)	Yes	All deviations involved Q1-Q3 estimates
Total (per model, 12 analyses overall)		624	48 (7.7)	Yes	All deviations involved Q1-Q3 estimates

For each dataset, ChatGPT and Claude produced identical results across all six analyses (two copy-paste analyses, two Excel-file analyses, and two Word-file analyses). Thus, each model completed 12 analyses overall across the two datasets. Each model was evaluated on 624 categories of descriptive statistics: 408 for auto.dta (68 categories × 6 analyses) and 216 for citytemp.dta (36 categories × 6 analyses). All deviations were observed identically across analyses and involved Q1–Q3 estimates.

### 3.1 Overall accuracy

ChatGPT and Claude produced identical results across all analyses. Across the two datasets, 104 distinct evaluable categories were assessed (68 for ‘auto.dta’ and 36 for ‘citytemp.dta’), of which eight (7.7%) differed from the Stata reference standard, corresponding to an overall accuracy of 92.3% (96/104 categories). Because each dataset was analyzed six times per model, a total of 624 category assessments were performed per model, of which 576 (92.3%) matched the Stata reference standard exactly.

No discrepancies were observed for the number of non-missing observations, number of missing observations, minimum and maximum values, means, standard deviations, medians, category frequencies, or category percentages. The only observed deviations involved first and third quartile (Q1–Q3) estimates for selected continuous variables.

### 3.2 Quartile deviations

The same eight Q1–Q3 deviations were observed for both models in every analysis. In the ‘auto.dta’ dataset, quartile estimates differed from the Stata reference values for the variables V2 (‘price’), V3 (‘mpg’), V6 (‘trunk’), V7 (‘weight’), V8 (‘length’), V10 (‘displacement’), and V11 (‘gear_ratio’). In the ‘citytemp.dta’ dataset, a quartile deviation was observed for V5 (‘tempjan’).

Post hoc analyses demonstrated that these discrepancies were entirely attributable to differences in quartile definitions rather than computational errors. When ChatGPT and Claude were explicitly instructed to use the same percentile definition as Stata's ‘codebook’ and ‘summarize, detail’ commands, the models reproduced the Stata Q1-Q3 values exactly for all affected variables. Conversely, when the models were instructed to use a linear interpolation approach, they reproduced exactly the quartile values observed in the primary analyses. For example, for V2 (‘price’) in ‘auto.dta’, the models generated the Stata values of 4195–6342 when instructed to use the Stata percentile definition and the values of 4220.25–6332.25 when instructed to use linear interpolation. Similar findings were observed for all other variables exhibiting quartile discrepancies.

### 3.3 Repeatability and between-modality consistency

Within-modality repeatability was complete for both models: identical results were obtained between the two independent repetitions for copy-paste, Microsoft Excel, and Microsoft Word inputs. Between-modality consistency was also complete, with identical results obtained across all three input modalities. Thus, all observed quartile deviations were reproduced consistently in each repetition and input modality for both models.

## 4 Discussion

### 4.1 Summary of findings

In this experimental study, ChatGPT and Claude demonstrated excellent performance in generating descriptive statistics from structured datasets. Across 624 evaluable categories per model, both LLMs produced identical results irrespective of input modality (copy-paste, Microsoft Word, or Microsoft Excel) and across repeated analyses. Exact agreement with the Stata reference standard was observed for 92.3% of evaluated categories.

All apparent discrepancies were limited to Q1-Q3 estimates for eight continuous variables; no deviations were observed for any other statistic. Furthermore, post hoc analyses demonstrated that the observed quartile differences resulted entirely from the use of a different quartile definition rather than computational errors. Consequently, no genuine calculation errors were identified for either ChatGPT or Claude for the descriptive statistics evaluated in this study; this finding should not be generalized to other mathematical or statistical tasks.

### 4.2 Comparison with existing literature

The present findings extend previous research evaluating the statistical capabilities of LLMs. Earlier studies reported promising performance for statistical reasoning, statistical test selection, statistical code generation, and sample-size estimation, although occasional numerical inaccuracies and reproducibility concerns have been described [10], [11], [12], [13], [21], [22]. Most previous investigations focused on statistical reasoning, inferential analyses, or software code generation rather than the direct computation of descriptive statistics from raw datasets. Reliability also varies across non-numerical research tasks. For example, Güneş et al. found substantial model-dependent differences in the accuracy of AI-generated scientific references, with fabricated references remaining an important concern for several evaluated LLMs [14]. In another structured clinical task, Çamur et al. reported high but model-dependent performance in applying CAD-RADS 2.0 criteria [16]. Together, these findings illustrate that LLM reliability is task- and model-dependent and that strong performance in one component of the biomedical workflow should not be assumed to generalize to others.

Only a limited number of studies have specifically examined descriptive statistical calculations. Ruta et al. reported that ChatGPT accurately generated means, standard deviations, medians, and interquartile ranges from an uploaded dataset; however, their study evaluated a single model and a single dataset, and did not compare performance across different dataset input modalities [13]. Similarly, Meo et al. evaluated ChatGPT using three biomedical datasets and a broad range of statistical tasks, including descriptive statistics, hypothesis testing, regression analyses, and graphical outputs. Although ChatGPT achieved an overall accuracy of 85.9%, the study was designed as a general assessment of biomedical statistical analysis rather than a focused evaluation of the accuracy and repeatability of descriptive statistical calculations or their consistency across different dataset input modalities [23].

The present study extends this literature by evaluating two contemporary LLMs, comparing performance across multiple dataset input modalities (copy-paste, Microsoft Word, and Microsoft Excel) and assessing within-modality repeatability and between-modality consistency. An additional contribution of our study concerns quartile estimation. At first glance, the observed Q1–Q3 discrepancies could have been interpreted as statistical errors. However, post hoc analyses demonstrated that the values generated by ChatGPT and Claude were mathematically correct under an alternative quartile definition based on linear interpolation. This finding highlights the importance of considering methodological conventions when evaluating AI-generated statistical outputs. Differences between statistical packages may reflect distinct computational definitions rather than genuine errors. This occurs because there is no single universally adopted algorithm for estimating quartiles from a finite sample. Different methods use different rules for selecting or interpolating between ordered observations and may therefore yield slightly different quartile values from the same dataset while remaining mathematically valid. Accordingly, agreement rates might differ if another statistical environment, such as R, SAS, Python, or SPSS, were used as the reference standard, particularly for statistics for which multiple valid computational conventions exist.

### 4.3 Implications for practice and research

The results suggest that ChatGPT and Claude may be useful tools for generating descriptive statistics in biomedical research. For relatively small and moderately sized datasets, both models accurately calculated missing-value counts, measures of central tendency, measures of dispersion, frequencies, and percentages, while demonstrating complete within-modality repeatability and between-modality consistency under the evaluated conditions.

These findings may be particularly relevant for researchers with limited access to statistical software or programming expertise. LLMs provide a natural-language interface that allows users to obtain descriptive statistics directly from pasted tables or uploaded files without writing code. Such functionality could facilitate exploratory analyses, educational activities, and preliminary data checking.

Nevertheless, caution remains warranted. First, our evaluation was limited to descriptive statistics and did not assess more advanced analytical procedures. Second, even when calculations are correct, differences in statistical conventions may produce results that differ from those generated by conventional software packages. Researchers should therefore verify which computational definitions are being used when exact agreement with a specific software package is required. More broadly, high numerical agreement in controlled benchmark tasks does not eliminate concerns related to opaque model processing, potential evaluation biases, or performance changes when models are applied to larger and more heterogeneous datasets. Standardized, model-agnostic, and independently auditable evaluation frameworks are therefore important for assessing LLM-generated outputs. Recent work, such as the MELMA framework, illustrates how structured, multidimensional assessment can complement aggregate performance measures when evaluating LLM-generated outputs [24]. Finally, despite the excellent performance observed in our study, AI-generated results should continue to be independently verified before being used in scientific publications or clinical research.

Future studies should evaluate larger datasets, more complex statistical procedures, additional LLMs, and newer model versions. Longitudinal studies should also assess reproducibility across successive model updates and compare proprietary and open-source LLMs. Further research could evaluate multimodal analytical workflows and end-to-end statistical pipelines integrating data cleaning, descriptive analysis, and inferential procedures, as well as performance in real-world datasets containing data-entry errors, missing data patterns, and more complex data structures than those examined in the present study. More broadly, LLM performance may vary across individual output parameters and task modalities, and aggregate accuracy measures may therefore obscure important differences in performance. Multidimensional evaluation across different output parameters and task modalities may provide a more comprehensive assessment of LLM performance than a single overall accuracy measure [25].

### 4.4 Limitations

Several limitations should be acknowledged. First, only two datasets were evaluated. Although the datasets differed substantially in size and variable composition, they were relatively clean benchmark datasets and may not fully represent the complexity and diversity of real-world datasets encountered in biomedical research. Clinical datasets may contain complex missing-data patterns, inconsistent formatting, duplicated variables, data-entry errors, and heterogeneous variable types, all of which could introduce additional challenges and potentially reduce LLM performance. Moreover, the largest dataset contained fewer than 1000 observations; therefore, the computational scalability of these models and their performance with datasets containing tens or hundreds of thousands of observations remain unknown. Second, our evaluation was limited to the direct computation of descriptive statistics. These tasks should be distinguished from higher-level statistical reasoning, such as test selection, assumption checking, hypothesis testing, regression modeling, survival analysis, or causal inference, for which LLM performance may differ substantially. Third, only three input modalities were examined. Other formats and data sources commonly encountered in biomedical research, including CSV files, PDFs, SPSS exports, REDCap exports, and SQL-derived tables, were not evaluated and may yield different results. Fourth, model temperature and other inference parameters were not controlled because the experiments were conducted through the standard user interfaces, and only two repetitions were performed per input modality. In addition, because the recorded outputs did not reveal the models' internal processing, we could not determine whether calculations were performed solely through model processing or with the assistance of embedded computational tools. The observed within-modality repeatability therefore provides limited evidence regarding stochastic variability and should not be interpreted as establishing reproducibility under repeated or controlled experimental conditions. Fifth, LLMs evolve rapidly, and our findings apply specifically to the evaluated versions (GPT-5.5 and Claude Sonnet 4.6). Future releases may differ in their architecture, computational tools, or underlying numerical procedures and may therefore produce different results. Finally, the post hoc analyses investigating quartile definitions were conducted after completion of the primary analyses. Although these analyses clearly explained all observed discrepancies, they were exploratory in nature and were not specified a priori.

## 5 Conclusions

ChatGPT and Claude demonstrated excellent accuracy and consistent results across repeated analyses and input modalities when generating descriptive statistics from structured datasets provided as copy-pasted tables, Microsoft Word documents, and Microsoft Excel files. Exact agreement with the Stata reference standard was observed for 92.3% of evaluated categories, and all apparent discrepancies were limited to quartile estimates. Post hoc analyses showed that these differences resulted from the use of an alternative, mathematically valid quartile definition rather than computational errors. After accounting for quartile methodology, no calculation errors were identified for either model across the 624 evaluated categories of descriptive statistics; these findings should not be generalized to other mathematical or statistical tasks.

Taken together, these results suggest that contemporary LLMs can accurately perform routine descriptive statistical analyses and may represent useful tools for data exploration, education, and research workflows. Nevertheless, the present study evaluated only two LLMs and two datasets. Further studies involving additional models, datasets, and statistical tasks are needed to determine the generalizability of these findings. Although the performance observed in this study was excellent, LLM-assisted statistical computation should complement rather than replace established statistical software and appropriate expert verification, particularly when analyses contribute to scientific publications or clinical decision-making.

## CRediT authorship contribution statement

**Paul Sebo:** Writing – original draft, Methodology, Investigation, Formal analysis, Data curation, Conceptualization. **Ting Wang:** Methodology, Investigation, Formal analysis, Data curation.

## Clinical trial number

Not applicable.

## Ethical approval

According to current Swiss legislation, ethical approval was not required because the study used publicly available datasets and did not involve human participants, biological material, or identifiable personal data.

## Declaration of Generative AI and AI-assisted technologies in the writing process

During the preparation of this manuscript, the authors used ChatGPT (OpenAI) to assist with language editing, grammar correction, readability improvements, and manuscript drafting. After using this tool, the authors carefully reviewed and edited all content and take full responsibility for the final published version of the manuscript.

## Funding

The authors received no specific funding for this work.

## Declaration of competing interest

The authors declare that they have no competing interests.

## Data Availability

All datasets used in this study are publicly available example datasets distributed with Stata and can be accessed using the commands ‘sysuse auto’ and ‘sysuse citytemp’. The prompts used in the primary and post hoc analyses are provided in Supplementary Material 1. Reference descriptive statistics generated with Stata are provided in Supplementary Material 2, and the complete outputs generated by ChatGPT and Claude are provided in Supplementary Materials 3 and 4.
