# Supplementary material for: Can large language models accurately compute descriptive statistics from structured datasets? A comparative evaluation of ChatGPT and Claude
Source: Am Heart J Plus. 2026 Aug 27;70:100877. doi: 10.1016/j.ahjo.2026.100877 (PMC13562389; doi:10.1016/j.ahjo.2026.100877)
Supplement: Supplementary file 1 [file mmc1.docx]

**Supplementary Material 1. Prompts used in the main study and post hoc quartile definition analyses (prompts 1a-6a: original prompts; prompts 1b-6b: Stata quartile definition; prompts 1c-6c: linear-interpolation quartile definition)**

**Prompt 1a – Database 1 (copy-paste)**

I will provide a dataset below. Please analyze the dataset and produce descriptive statistics for all variables according to the instructions provided. Report all numerical results with two decimal places when applicable. Do not provide explanations, interpretations, or comments. Present the results in clear tables.

Ignore variable V1 entirely. For variables V4, V5, and V12, report the number of non-missing observations, the number of missing observations, the frequency of each category, and the percentage of each category calculated using non-missing observations as the denominator. For variables V2, V3, V6, V7, V8, V9, V10, and V11, report the number of non-missing observations, the number of missing observations, the minimum, the maximum, the mean, the standard deviation, the median, the first quartile (Q1), and the third quartile (Q3).

Please analyze the dataset as provided below.

[PASTE DATASET HERE]

**Prompt 2a – Database 1 (Excel)**

An Excel file containing a dataset is attached. Please analyze the dataset and produce descriptive statistics for all variables according to the instructions provided. Report all numerical results with two decimal places when applicable. Do not provide explanations, interpretations, or comments. Present the results in clear tables.

Ignore variable V1 entirely. For variables V4, V5, and V12, report the number of non-missing observations, the number of missing observations, the frequency of each category, and the percentage of each category calculated using non-missing observations as the denominator. For variables V2, V3, V6, V7, V8, V9, V10, and V11, report the number of non-missing observations, the number of missing observations, the minimum, the maximum, the mean, the standard deviation, the median, the first quartile (Q1), and the third quartile (Q3).

Please analyze the attached Excel dataset.

**Prompt 3a – Database 1 (Word)**

A Word document containing a dataset is attached. Please analyze the dataset and produce descriptive statistics for all variables according to the instructions provided. Report all numerical results with two decimal places when applicable. Do not provide explanations, interpretations, or comments. Present the results in clear tables.

Ignore variable V1 entirely. For variables V4, V5, and V12, report the number of non-missing observations, the number of missing observations, the frequency of each category, and the percentage of each category calculated using non-missing observations as the denominator. For variables V2, V3, V6, V7, V8, V9, V10, and V11, report the number of non-missing observations, the number of missing observations, the minimum, the maximum, the mean, the standard deviation, the median, the first quartile (Q1), and the third quartile (Q3).

Please analyze the attached Word dataset.

**Prompt 4a – Database 2 (copy-paste)**

I will provide a dataset below. Please analyze the dataset and produce descriptive statistics for all variables according to the instructions provided. Report all numerical results with two decimal places when applicable. Do not provide explanations, interpretations, or comments. Present the results in clear tables.

For variables V1 and V2, report the number of non-missing observations, the number of missing observations, the frequency of each category, and the percentage of each category calculated using non-missing observations as the denominator. For variables V3, V4, V5, and V6, report the number of non-missing observations, the number of missing observations, the minimum, the maximum, the mean, the standard deviation, the median, the first quartile (Q1), and the third quartile (Q3).

Please analyze the dataset as provided below.

[PASTE DATASET HERE]

**Prompt 5a – Database 2 (Excel)**

An Excel file containing a dataset is attached. Please analyze the dataset and produce descriptive statistics for all variables according to the instructions provided. Report all numerical results with two decimal places when applicable. Do not provide explanations, interpretations, or comments. Present the results in clear tables.

For variables V1 and V2, report the number of non-missing observations, the number of missing observations, the frequency of each category, and the percentage of each category calculated using non-missing observations as the denominator. For variables V3, V4, V5, and V6, report the number of non-missing observations, the number of missing observations, the minimum, the maximum, the mean, the standard deviation, the median, the first quartile (Q1), and the third quartile (Q3).

Please analyze the attached Excel dataset.

**Prompt 6a – Database 2 (Word)**

A Word document containing a dataset is attached. Please analyze the dataset and produce descriptive statistics for all variables according to the instructions provided. Report all numerical results with two decimal places when applicable. Do not provide explanations, interpretations, or comments. Present the results in clear tables.

For variables V1 and V2, report the number of non-missing observations, the number of missing observations, the frequency of each category, and the percentage of each category calculated using non-missing observations as the denominator. For variables V3, V4, V5, and V6, report the number of non-missing observations, the number of missing observations, the minimum, the maximum, the mean, the standard deviation, the median, the first quartile (Q1), and the third quartile (Q3).

Please analyze the attached Word dataset.

**Prompt 1b – Database 1 (copy-paste)**

I will provide a dataset below. Please analyze the dataset and produce descriptive statistics for all variables according to the instructions provided. Report all numerical results with two decimal places when applicable. Do not provide explanations, interpretations, or comments. Present the results in clear tables.

Ignore variable V1 entirely. For variables V4, V5, and V12, report the number of non-missing observations, the number of missing observations, the frequency of each category, and the percentage of each category calculated using non-missing observations as the denominator. For variables V2, V3, V6, V7, V8, V9, V10, and V11, report the number of non-missing observations, the number of missing observations, the minimum, the maximum, the mean, the standard deviation, the median, the first quartile (Q1), and the third quartile (Q3).

For Q1 and Q3, use the same quartile definition as Stata's codebook and summarize, detail commands. Specifically, for a dataset with n non-missing observations and percentile p, calculate P = n × p. If P is an integer, the percentile is the average of the observations at ranks P and P+1 in the sorted data. If P is not an integer, the percentile is the observation at the first rank strictly greater than P. Do not use linear interpolation between observations.

Please analyze the dataset as provided below.

[PASTE DATASET HERE]

**Prompt 2b – Database 1 (Excel)**

An Excel file containing a dataset is attached. Please analyze the dataset and produce descriptive statistics for all variables according to the instructions provided. Report all numerical results with two decimal places when applicable. Do not provide explanations, interpretations, or comments. Present the results in clear tables.

Ignore variable V1 entirely. For variables V4, V5, and V12, report the number of non-missing observations, the number of missing observations, the frequency of each category, and the percentage of each category calculated using non-missing observations as the denominator. For variables V2, V3, V6, V7, V8, V9, V10, and V11, report the number of non-missing observations, the number of missing observations, the minimum, the maximum, the mean, the standard deviation, the median, the first quartile (Q1), and the third quartile (Q3).

For Q1 and Q3, use the same quartile definition as Stata's codebook and summarize, detail commands. Specifically, for a dataset with n non-missing observations and percentile p, calculate P = n × p. If P is an integer, the percentile is the average of the observations at ranks P and P+1 in the sorted data. If P is not an integer, the percentile is the observation at the first rank strictly greater than P. Do not use linear interpolation between observations.

Please analyze the attached Excel dataset.

**Prompt 3b – Database 1 (Word)**

A Word document containing a dataset is attached. Please analyze the dataset and produce descriptive statistics for all variables according to the instructions provided. Report all numerical results with two decimal places when applicable. Do not provide explanations, interpretations, or comments. Present the results in clear tables.

Ignore variable V1 entirely. For variables V4, V5, and V12, report the number of non-missing observations, the number of missing observations, the frequency of each category, and the percentage of each category calculated using non-missing observations as the denominator. For variables V2, V3, V6, V7, V8, V9, V10, and V11, report the number of non-missing observations, the number of missing observations, the minimum, the maximum, the mean, the standard deviation, the median, the first quartile (Q1), and the third quartile (Q3).

For Q1 and Q3, use the same quartile definition as Stata's codebook and summarize, detail commands. Specifically, for a dataset with n non-missing observations and percentile p, calculate P = n × p. If P is an integer, the percentile is the average of the observations at ranks P and P+1 in the sorted data. If P is not an integer, the percentile is the observation at the first rank strictly greater than P. Do not use linear interpolation between observations.

Please analyze the attached Word dataset.

**Prompt 4b – Database 2 (copy-paste)**

I will provide a dataset below. Please analyze the dataset and produce descriptive statistics for all variables according to the instructions provided. Report all numerical results with two decimal places when applicable. Do not provide explanations, interpretations, or comments. Present the results in clear tables.

For variables V1 and V2, report the number of non-missing observations, the number of missing observations, the frequency of each category, and the percentage of each category calculated using non-missing observations as the denominator. For variables V3, V4, V5, and V6, report the number of non-missing observations, the number of missing observations, the minimum, the maximum, the mean, the standard deviation, the median, the first quartile (Q1), and the third quartile (Q3).

For Q1 and Q3, use the same quartile definition as Stata's codebook and summarize, detail commands. Specifically, for a dataset with n non-missing observations and percentile p, calculate P = n × p. If P is an integer, the percentile is the average of the observations at ranks P and P+1 in the sorted data. If P is not an integer, the percentile is the observation at the first rank strictly greater than P. Do not use linear interpolation between observations.

Please analyze the dataset as provided below.

[PASTE DATASET HERE]

**Prompt 5b – Database 2 (Excel)**

An Excel file containing a dataset is attached. Please analyze the dataset and produce descriptive statistics for all variables according to the instructions provided. Report all numerical results with two decimal places when applicable. Do not provide explanations, interpretations, or comments. Present the results in clear tables.

For variables V1 and V2, report the number of non-missing observations, the number of missing observations, the frequency of each category, and the percentage of each category calculated using non-missing observations as the denominator. For variables V3, V4, V5, and V6, report the number of non-missing observations, the number of missing observations, the minimum, the maximum, the mean, the standard deviation, the median, the first quartile (Q1), and the third quartile (Q3).

For Q1 and Q3, use the same quartile definition as Stata's codebook and summarize, detail commands. Specifically, for a dataset with n non-missing observations and percentile p, calculate P = n × p. If P is an integer, the percentile is the average of the observations at ranks P and P+1 in the sorted data. If P is not an integer, the percentile is the observation at the first rank strictly greater than P. Do not use linear interpolation between observations.

Please analyze the attached Excel dataset.

**Prompt 6b – Database 2 (Word)**

A Word document containing a dataset is attached. Please analyze the dataset and produce descriptive statistics for all variables according to the instructions provided. Report all numerical results with two decimal places when applicable. Do not provide explanations, interpretations, or comments. Present the results in clear tables.

For variables V1 and V2, report the number of non-missing observations, the number of missing observations, the frequency of each category, and the percentage of each category calculated using non-missing observations as the denominator. For variables V3, V4, V5, and V6, report the number of non-missing observations, the number of missing observations, the minimum, the maximum, the mean, the standard deviation, the median, the first quartile (Q1), and the third quartile (Q3).

For Q1 and Q3, use the same quartile definition as Stata's codebook and summarize, detail commands. Specifically, for a dataset with n non-missing observations and percentile p, calculate P = n × p. If P is an integer, the percentile is the average of the observations at ranks P and P+1 in the sorted data. If P is not an integer, the percentile is the observation at the first rank strictly greater than P. Do not use linear interpolation between observations.

Please analyze the attached Word dataset.

**Prompt 1c – Database 1 (copy-paste)**

I will provide a dataset below. Please analyze the dataset and produce descriptive statistics for all variables according to the instructions provided. Report all numerical results with two decimal places when applicable. Do not provide explanations, interpretations, or comments. Present the results in clear tables.

Ignore variable V1 entirely. For variables V4, V5, and V12, report the number of non-missing observations, the number of missing observations, the frequency of each category, and the percentage of each category calculated using non-missing observations as the denominator. For variables V2, V3, V6, V7, V8, V9, V10, and V11, report the number of non-missing observations, the number of missing observations, the minimum, the maximum, the mean, the standard deviation, the median, the first quartile (Q1), and the third quartile (Q3).

For Q1 and Q3, use linear interpolation. Specifically, for a variable with n non-missing observations and percentile p, first sort the non-missing values in ascending order. Then calculate the percentile position as h = 1 + (n − 1) × p, where p = 0.25 for Q1 and p = 0.75 for Q3. If h is an integer, the percentile is the value at rank h. If h is not an integer, let k be the integer part of h and d = h − k; the percentile is calculated as x(k) + d × [x(k+1) − x(k)], where x(k) and x(k+1) are the sorted values at ranks k and k+1. Do not use Stata's default adjacent-order-statistic method for Q1 and Q3.

Please analyze the dataset as provided below.

[PASTE DATASET HERE]

**Prompt 2c – Database 1 (Excel)**

An Excel file containing a dataset is attached. Please analyze the dataset and produce descriptive statistics for all variables according to the instructions provided. Report all numerical results with two decimal places when applicable. Do not provide explanations, interpretations, or comments. Present the results in clear tables.

Ignore variable V1 entirely. For variables V4, V5, and V12, report the number of non-missing observations, the number of missing observations, the frequency of each category, and the percentage of each category calculated using non-missing observations as the denominator. For variables V2, V3, V6, V7, V8, V9, V10, and V11, report the number of non-missing observations, the number of missing observations, the minimum, the maximum, the mean, the standard deviation, the median, the first quartile (Q1), and the third quartile (Q3).

For Q1 and Q3, use linear interpolation. Specifically, for a variable with n non-missing observations and percentile p, first sort the non-missing values in ascending order. Then calculate the percentile position as h = 1 + (n − 1) × p, where p = 0.25 for Q1 and p = 0.75 for Q3. If h is an integer, the percentile is the value at rank h. If h is not an integer, let k be the integer part of h and d = h − k; the percentile is calculated as x(k) + d × [x(k+1) − x(k)], where x(k) and x(k+1) are the sorted values at ranks k and k+1. Do not use Stata's default adjacent-order-statistic method for Q1 and Q3.

Please analyze the attached Excel dataset.

**Prompt 3c – Database 1 (Word)**

A Word document containing a dataset is attached. Please analyze the dataset and produce descriptive statistics for all variables according to the instructions provided. Report all numerical results with two decimal places when applicable. Do not provide explanations, interpretations, or comments. Present the results in clear tables.

Ignore variable V1 entirely. For variables V4, V5, and V12, report the number of non-missing observations, the number of missing observations, the frequency of each category, and the percentage of each category calculated using non-missing observations as the denominator. For variables V2, V3, V6, V7, V8, V9, V10, and V11, report the number of non-missing observations, the number of missing observations, the minimum, the maximum, the mean, the standard deviation, the median, the first quartile (Q1), and the third quartile (Q3).

For Q1 and Q3, use linear interpolation. Specifically, for a variable with n non-missing observations and percentile p, first sort the non-missing values in ascending order. Then calculate the percentile position as h = 1 + (n − 1) × p, where p = 0.25 for Q1 and p = 0.75 for Q3. If h is an integer, the percentile is the value at rank h. If h is not an integer, let k be the integer part of h and d = h − k; the percentile is calculated as x(k) + d × [x(k+1) − x(k)], where x(k) and x(k+1) are the sorted values at ranks k and k+1. Do not use Stata's default adjacent-order-statistic method for Q1 and Q3.

Please analyze the attached Word dataset.

**Prompt 4c – Database 2 (copy-paste)**

I will provide a dataset below. Please analyze the dataset and produce descriptive statistics for all variables according to the instructions provided. Report all numerical results with two decimal places when applicable. Do not provide explanations, interpretations, or comments. Present the results in clear tables.

For variables V1 and V2, report the number of non-missing observations, the number of missing observations, the frequency of each category, and the percentage of each category calculated using non-missing observations as the denominator. For variables V3, V4, V5, and V6, report the number of non-missing observations, the number of missing observations, the minimum, the maximum, the mean, the standard deviation, the median, the first quartile (Q1), and the third quartile (Q3).

For Q1 and Q3, use linear interpolation. Specifically, for a variable with n non-missing observations and percentile p, first sort the non-missing values in ascending order. Then calculate the percentile position as h = 1 + (n − 1) × p, where p = 0.25 for Q1 and p = 0.75 for Q3. If h is an integer, the percentile is the value at rank h. If h is not an integer, let k be the integer part of h and d = h − k; the percentile is calculated as x(k) + d × [x(k+1) − x(k)], where x(k) and x(k+1) are the sorted values at ranks k and k+1. Do not use Stata's default adjacent-order-statistic method for Q1 and Q3.

Please analyze the dataset as provided below.

[PASTE DATASET HERE]

**Prompt 5c – Database 2 (Excel)**

An Excel file containing a dataset is attached. Please analyze the dataset and produce descriptive statistics for all variables according to the instructions provided. Report all numerical results with two decimal places when applicable. Do not provide explanations, interpretations, or comments. Present the results in clear tables.

For variables V1 and V2, report the number of non-missing observations, the number of missing observations, the frequency of each category, and the percentage of each category calculated using non-missing observations as the denominator. For variables V3, V4, V5, and V6, report the number of non-missing observations, the number of missing observations, the minimum, the maximum, the mean, the standard deviation, the median, the first quartile (Q1), and the third quartile (Q3).

For Q1 and Q3, use linear interpolation. Specifically, for a variable with n non-missing observations and percentile p, first sort the non-missing values in ascending order. Then calculate the percentile position as h = 1 + (n − 1) × p, where p = 0.25 for Q1 and p = 0.75 for Q3. If h is an integer, the percentile is the value at rank h. If h is not an integer, let k be the integer part of h and d = h − k; the percentile is calculated as x(k) + d × [x(k+1) − x(k)], where x(k) and x(k+1) are the sorted values at ranks k and k+1. Do not use Stata's default adjacent-order-statistic method for Q1 and Q3.

Please analyze the attached Excel dataset.

**Prompt 6c – Database 2 (Word)**

A Word document containing a dataset is attached. Please analyze the dataset and produce descriptive statistics for all variables according to the instructions provided. Report all numerical results with two decimal places when applicable. Do not provide explanations, interpretations, or comments. Present the results in clear tables.

For variables V1 and V2, report the number of non-missing observations, the number of missing observations, the frequency of each category, and the percentage of each category calculated using non-missing observations as the denominator. For variables V3, V4, V5, and V6, report the number of non-missing observations, the number of missing observations, the minimum, the maximum, the mean, the standard deviation, the median, the first quartile (Q1), and the third quartile (Q3).

For Q1 and Q3, use linear interpolation. Specifically, for a variable with n non-missing observations and percentile p, first sort the non-missing values in ascending order. Then calculate the percentile position as h = 1 + (n − 1) × p, where p = 0.25 for Q1 and p = 0.75 for Q3. If h is an integer, the percentile is the value at rank h. If h is not an integer, let k be the integer part of h and d = h − k; the percentile is calculated as x(k) + d × [x(k+1) − x(k)], where x(k) and x(k+1) are the sorted values at ranks k and k+1. Do not use Stata's default adjacent-order-statistic method for Q1 and Q3.

Please analyze the attached Word dataset.

**Supplementary Material 2. Reference descriptive statistics for the two datasets**

| Variable | Original variable name | Non-missing observations, n | Missing observations, n | Min-Max | Mean | SD | Median | Q1-Q3 | Category frequencies, n | Category frequencies, % |
| --- | --- | --- | --- | --- | --- | --- | --- | --- | --- | --- |
| auto.dta |  |  |  |  |  |  |  |  |  |  |
| V1 | make | N/A | N/A | N/A | N/A | N/A | N/A | N/A | N/A | N/A |
| V2 | price | 74 | 0 | 3291-15906 | 6165.26 | 2949.5 | 5006.5 | 4195-6342 | N/A | N/A |
| V3 | mpg | 74 | 0 | 12-41 | 21.30 | 5.79 | 20 | 18-25 | NA | NA |
| V4 | rep78 | 69 | 5 | NA | NA | NA | NA | NA | “1”=2, “2”=8, “3”=30, “4”=18, “5”=11 | “1”=2.90, “2”=11.59, “3”=43.48, “4”=26.09, “5”=15.94 |
| V5 | headroom | 74 | 0 | NA | NA | NA | NA | NA | “1.5”=4, “2”=13, “2.5”=14, “3”=13, “3.5”=15, “4”=10, “4.5”=4, “5”=1 | “1.5”=5.41, “2”=17.57, “2.5”=18.92, “3”=17.57, “3.5”=20.27, “4”=13.51, “4.5”=5.41, “5”=1.35 |
| V6 | trunk | 74 | 0 | 5-23 | 13.76 | 4.28 | 14 | 10-17 | N/A | N/A |
| V7 | weight | 74 | 0 | 1760-4840 | 3019.46 | 777.19 | 3190 | 2240-3600 | N/A | N/A |
| V8 | length | 74 | 0 | 142-233 | 187.93 | 22.27 | 192.5 | 170-204 | N/A | N/A |
| V9 | turn | 74 | 0 | 31-51 | 39.65 | 4.40 | 40 | 36-43 | N/A | N/A |
| V10 | displacement | 74 | 0 | 79-425 | 197.30 | 91.84 | 196 | 119-250 | N/A | N/A |
| V11 | gear_ratio | 74 | 0 | 2.19-3.89 | 3.01 | 0.46 | 2.96 | 2.73-3.37 | N/A | N/A |
| V12 | foreign | 74 | 0 | N/A | N/A | N/A | N/A | N/A | “0”=52, “1”=22 | “0”=70.27, “1”=29.73 |
| citytemp.dta |  |  |  |  |  |  |  |  |  |  |
| V1 | division | 956 | 0 | N/A | N/A | N/A | N/A | N/A | “1”=69, “2”=97, “3”=206, “4”=78, “5”=115, “6”=46, “7”=89, “8”=61, “9”=195 | “1”=7.22, “2”=10.15, “3”=21.55, “4”=8.16, “5”=12.03, “6”=4.81, “7”=9.31, “8”=6.38, “9”=20.40 |
| V2 | region | 956 | 0 | N/A | N/A | N/A | N/A | N/A | “1”=166, “2”=284, “3”=250, “4”=256 | “1”=17.36, “2”=29.71, “3”=26.15, “4”=26.78 |
| V3 | heatdd | 953 | 3 | 0-10816 | 4425.53 | 2199.6 | 4950 | 2460-6232 | N/A | N/A |
| V4 | cooldd | 953 | 3 | 0-4389 | 1240.41 | 937.67 | 940 | 615-1566 | N/A | N/A |
| V5 | tempjan | 954 | 2 | 2.2-72.6 | 35.75 | 14.19 | 31.3 | 25.1-47.8 | N/A | N/A |
| V6 | tempjuly | 954 | 2 | 58.1-93.6 | 75.05 | 5.50 | 74.25 | 71.8-78.7 | N/A | N/A |

**Supplementary Material 3. ChatGPT-generated descriptive statistics for the ‘auto.dta’ and ‘citytemp.dta’ datasets across input formats and repeated runs**

| Variable | Original variable name | Non-missing observations, n | Missing observations, n | Min-Max | Mean | SD | Median | Q1-Q3 | Category frequencies, n | Category frequencies, % |
| --- | --- | --- | --- | --- | --- | --- | --- | --- | --- | --- |
| auto.dta |  |  |  |  |  |  |  |  |  |  |
| Copy paste |  |  |  |  |  |  |  |  |  |  |
| V1 – round 1 | make | N/A | N/A | N/A | N/A | N/A | N/A | N/A | N/A | N/A |
| V1 – round 2 | make | N/A | N/A | N/A | N/A | N/A | N/A | N/A | N/A | N/A |
| V2 – round 1 | price | 74 | 0 | 3291-15906 | 6165.26 | 2949.5 | 5006.5 | 4220.25-6332.25 | N/A | N/A |
| V2 – round 2 | price | 74 | 0 | 3291-15906 | 6165.26 | 2949.5 | 5006.5 | 4220.25-6332.25 | N/A | N/A |
| V3 – round 1 | mpg | 74 | 0 | 12-41 | 21.30 | 5.79 | 20 | 18-24.75 | NA | NA |
| V3 – round 2 | mpg | 74 | 0 | 12-41 | 21.30 | 5.79 | 20 | 18-24.75 | NA | NA |
| V4 – round 1 | rep78 | 69 | 5 | NA | NA | NA | NA | NA | “1”=2, “2”=8, “3”=30, “4”=18, “5”=11 | “1”=2.90, “2”=11.59, “3”=43.48, “4”=26.09, “5”=15.94 |
| V4 – round 2 | rep78 | 69 | 5 | NA | NA | NA | NA | NA | “1”=2, “2”=8, “3”=30, “4”=18, “5”=11 | “1”=2.90, “2”=11.59, “3”=43.48, “4”=26.09, “5”=15.94 |
| V5 – round 1 | headroom | 74 | 0 | NA | NA | NA | NA | NA | “1.5”=4, “2”=13, “2.5”=14, “3”=13, “3.5”=15, “4”=10, “4.5”=4, “5”=1 | “1.5”=5.41, “2”=17.57, “2.5”=18.92, “3”=17.57, “3.5”=20.27, “4”=13.51, “4.5”=5.41, “5”=1.35 |
| V5 – round 2 | headroom | 74 | 0 | NA | NA | NA | NA | NA | “1.5”=4, “2”=13, “2.5”=14, “3”=13, “3.5”=15, “4”=10, “4.5”=4, “5”=1 | “1.5”=5.41, “2”=17.57, “2.5”=18.92, “3”=17.57, “3.5”=20.27, “4”=13.51, “4.5”=5.41, “5”=1.35 |
| V6 – round 1 | trunk | 74 | 0 | 5-23 | 13.76 | 4.28 | 14 | 10.25-16.75 | N/A | N/A |
| V6 – round 2 | trunk | 74 | 0 | 5-23 | 13.76 | 4.28 | 14 | 10.25-16.75 | N/A | N/A |
| V7 – round 1 | weight | 74 | 0 | 1760-4840 | 3019.46 | 777.19 | 3190 | 2250-3600 | N/A | N/A |
| V7 – round 2 | weight | 74 | 0 | 1760-4840 | 3019.46 | 777.19 | 3190 | 2250-3600 | N/A | N/A |
| V8 – round 1 | length | 74 | 0 | 142-233 | 187.93 | 22.27 | 192.5 | 170-203.75 | N/A | N/A |
| V8 – round 2 | length | 74 | 0 | 142-233 | 187.93 | 22.27 | 192.5 | 170-203.75 | N/A | N/A |
| V9 – round 1 | turn | 74 | 0 | 31-51 | 39.65 | 4.40 | 40 | 36-43 | N/A | N/A |
| V9 – round 2 | turn | 74 | 0 | 31-51 | 39.65 | 4.40 | 40 | 36-43 | N/A | N/A |
| V10 – round 1 | displacement | 74 | 0 | 79-425 | 197.30 | 91.84 | 196 | 119-245.25 | N/A | N/A |
| V10 – round 2 | displacement | 74 | 0 | 79-425 | 197.30 | 91.84 | 196 | 119-245.25 | N/A | N/A |
| V11 – round 1 | gear_ratio | 74 | 0 | 2.19-3.89 | 3.01 | 0.46 | 2.96 | 2.73-3.35 | N/A | N/A |
| V11 – round 2 | gear_ratio | 74 | 0 | 2.19-3.89 | 3.01 | 0.46 | 2.96 | 2.73-3.35 | N/A | N/A |
| V12 – round 1 | foreign | 74 | 0 | N/A | N/A | N/A | N/A | N/A | “0”=52, “1”=22 | “0”=70.27, “1”=29.73 |
| V12 – round 2 | foreign | 74 | 0 | N/A | N/A | N/A | N/A | N/A | “0”=52, “1”=22 | “0”=70.27, “1”=29.73 |
| Excel |  |  |  |  |  |  |  |  |  |  |
| V1 – round 1 | make | N/A | N/A | N/A | N/A | N/A | N/A | N/A | N/A | N/A |
| V1 – round 2 | make | N/A | N/A | N/A | N/A | N/A | N/A | N/A | N/A | N/A |
| V2 – round 1 | price | 74 | 0 | 3291-15906 | 6165.26 | 2949.5 | 5006.5 | 4220.25-6332.25 | N/A | N/A |
| V2 – round 2 | price | 74 | 0 | 3291-15906 | 6165.26 | 2949.5 | 5006.5 | 4220.25-6332.25 | N/A | N/A |
| V3 – round 1 | mpg | 74 | 0 | 12-41 | 21.30 | 5.79 | 20 | 18-24.75 | NA | NA |
| V3 – round 2 | mpg | 74 | 0 | 12-41 | 21.30 | 5.79 | 20 | 18-24.75 | NA | NA |
| V4 – round 1 | rep78 | 69 | 5 | NA | NA | NA | NA | NA | “1”=2, “2”=8, “3”=30, “4”=18, “5”=11 | “1”=2.90, “2”=11.59, “3”=43.48, “4”=26.09, “5”=15.94 |
| V4 – round 2 | rep78 | 69 | 5 | NA | NA | NA | NA | NA | “1”=2, “2”=8, “3”=30, “4”=18, “5”=11 | “1”=2.90, “2”=11.59, “3”=43.48, “4”=26.09, “5”=15.94 |
| V5 – round 1 | headroom | 74 | 0 | NA | NA | NA | NA | NA | “1.5”=4, “2”=13, “2.5”=14, “3”=13, “3.5”=15, “4”=10, “4.5”=4, “5”=1 | “1.5”=5.41, “2”=17.57, “2.5”=18.92, “3”=17.57, “3.5”=20.27, “4”=13.51, “4.5”=5.41, “5”=1.35 |
| V5 – round 2 | headroom | 74 | 0 | NA | NA | NA | NA | NA | “1.5”=4, “2”=13, “2.5”=14, “3”=13, “3.5”=15, “4”=10, “4.5”=4, “5”=1 | “1.5”=5.41, “2”=17.57, “2.5”=18.92, “3”=17.57, “3.5”=20.27, “4”=13.51, “4.5”=5.41, “5”=1.35 |
| V6 – round 1 | trunk | 74 | 0 | 5-23 | 13.76 | 4.28 | 14 | 10.25-16.75 | N/A | N/A |
| V6 – round 2 | trunk | 74 | 0 | 5-23 | 13.76 | 4.28 | 14 | 10.25-16.75 | N/A | N/A |
| V7 – round 1 | weight | 74 | 0 | 1760-4840 | 3019.46 | 777.19 | 3190 | 2250-3600 | N/A | N/A |
| V7 – round 2 | weight | 74 | 0 | 1760-4840 | 3019.46 | 777.19 | 3190 | 2250-3600 | N/A | N/A |
| V8 – round 1 | length | 74 | 0 | 142-233 | 187.93 | 22.27 | 192.5 | 170-203.75 | N/A | N/A |
| V8 – round 2 | length | 74 | 0 | 142-233 | 187.93 | 22.27 | 192.5 | 170-203.75 | N/A | N/A |
| V9 – round 1 | turn | 74 | 0 | 31-51 | 39.65 | 4.40 | 40 | 36-43 | N/A | N/A |
| V9 – round 2 | turn | 74 | 0 | 31-51 | 39.65 | 4.40 | 40 | 36-43 | N/A | N/A |
| V10 – round 1 | displacement | 74 | 0 | 79-425 | 197.30 | 91.84 | 196 | 119-245.25 | N/A | N/A |
| V10 – round 2 | displacement | 74 | 0 | 79-425 | 197.30 | 91.84 | 196 | 119-245.25 | N/A | N/A |
| V11 – round 1 | gear_ratio | 74 | 0 | 2.19-3.89 | 3.01 | 0.46 | 2.96 | 2.73-3.35 | N/A | N/A |
| V11 – round 2 | gear_ratio | 74 | 0 | 2.19-3.89 | 3.01 | 0.46 | 2.96 | 2.73-3.35 | N/A | N/A |
| V12 – round 1 | foreign | 74 | 0 | N/A | N/A | N/A | N/A | N/A | “0”=52, “1”=22 | “0”=70.27, “1”=29.73 |
| V12 – round 2 | foreign | 74 | 0 | N/A | N/A | N/A | N/A | N/A | “0”=52, “1”=22 | “0”=70.27, “1”=29.73 |
| Word |  |  |  |  |  |  |  |  |  |  |
| V1 – round 1 | make | N/A | N/A | N/A | N/A | N/A | N/A | N/A | N/A | N/A |
| V1 – round 2 | make | N/A | N/A | N/A | N/A | N/A | N/A | N/A | N/A | N/A |
| V2 – round 1 | price | 74 | 0 | 3291-15906 | 6165.26 | 2949.5 | 5006.5 | 4220.25-6332.25 | N/A | N/A |
| V2 – round 2 | price | 74 | 0 | 3291-15906 | 6165.26 | 2949.5 | 5006.5 | 4220.25-6332.25 | N/A | N/A |
| V3 – round 1 | mpg | 74 | 0 | 12-41 | 21.30 | 5.79 | 20 | 18-24.75 | NA | NA |
| V3 – round 2 | mpg | 74 | 0 | 12-41 | 21.30 | 5.79 | 20 | 18-24.75 | NA | NA |
| V4 – round 1 | rep78 | 69 | 5 | NA | NA | NA | NA | NA | “1”=2, “2”=8, “3”=30, “4”=18, “5”=11 | “1”=2.90, “2”=11.59, “3”=43.48, “4”=26.09, “5”=15.94 |
| V4 – round 2 | rep78 | 69 | 5 | NA | NA | NA | NA | NA | “1”=2, “2”=8, “3”=30, “4”=18, “5”=11 | “1”=2.90, “2”=11.59, “3”=43.48, “4”=26.09, “5”=15.94 |
| V5 – round 1 | headroom | 74 | 0 | NA | NA | NA | NA | NA | “1.5”=4, “2”=13, “2.5”=14, “3”=13, “3.5”=15, “4”=10, “4.5”=4, “5”=1 | “1.5”=5.41, “2”=17.57, “2.5”=18.92, “3”=17.57, “3.5”=20.27, “4”=13.51, “4.5”=5.41, “5”=1.35 |
| V5 – round 2 | headroom | 74 | 0 | NA | NA | NA | NA | NA | “1.5”=4, “2”=13, “2.5”=14, “3”=13, “3.5”=15, “4”=10, “4.5”=4, “5”=1 | “1.5”=5.41, “2”=17.57, “2.5”=18.92, “3”=17.57, “3.5”=20.27, “4”=13.51, “4.5”=5.41, “5”=1.35 |
| V6 – round 1 | trunk | 74 | 0 | 5-23 | 13.76 | 4.28 | 14 | 10.25-16.75 | N/A | N/A |
| V6 – round 2 | trunk | 74 | 0 | 5-23 | 13.76 | 4.28 | 14 | 10.25-16.75 | N/A | N/A |
| V7 – round 1 | weight | 74 | 0 | 1760-4840 | 3019.46 | 777.19 | 3190 | 2250-3600 | N/A | N/A |
| V7 – round 2 | weight | 74 | 0 | 1760-4840 | 3019.46 | 777.19 | 3190 | 2250-3600 | N/A | N/A |
| V8 – round 1 | length | 74 | 0 | 142-233 | 187.93 | 22.27 | 192.5 | 170-203.75 | N/A | N/A |
| V8 – round 2 | length | 74 | 0 | 142-233 | 187.93 | 22.27 | 192.5 | 170-203.75 | N/A | N/A |
| V9 – round 1 | turn | 74 | 0 | 31-51 | 39.65 | 4.40 | 40 | 36-43 | N/A | N/A |
| V9 – round 2 | turn | 74 | 0 | 31-51 | 39.65 | 4.40 | 40 | 36-43 | N/A | N/A |
| V10 – round 1 | displacement | 74 | 0 | 79-425 | 197.30 | 91.84 | 196 | 119-245.25 | N/A | N/A |
| V10 – round 2 | displacement | 74 | 0 | 79-425 | 197.30 | 91.84 | 196 | 119-245.25 | N/A | N/A |
| V11 – round 1 | gear_ratio | 74 | 0 | 2.19-3.89 | 3.01 | 0.46 | 2.96 | 2.73-3.35 | N/A | N/A |
| V11 – round 2 | gear_ratio | 74 | 0 | 2.19-3.89 | 3.01 | 0.46 | 2.96 | 2.73-3.35 | N/A | N/A |
| V12 – round 1 | foreign | 74 | 0 | N/A | N/A | N/A | N/A | N/A | “0”=52, “1”=22 | “0”=70.27, “1”=29.73 |
| V12 – round 2 | foreign | 74 | 0 | N/A | N/A | N/A | N/A | N/A | “0”=52, “1”=22 | “0”=70.27, “1”=29.73 |
| citytemp.dta |  |  |  |  |  |  |  |  |  |  |
| Copy paste |  |  |  |  |  |  |  |  |  |  |
| V1 – round 1 | division | 956 | 0 | N/A | N/A | N/A | N/A | N/A | “1”=69, “2”=97, “3”=206, “4”=78, “5”=115, “6”=46, “7”=89, “8”=61, “9”=195 | “1”=7.22, “2”=10.15, “3”=21.55, “4”=8.16, “5”=12.03, “6”=4.81, “7”=9.31, “8”=6.38, “9”=20.40 |
| V1 – round 2 | division | 956 | 0 | N/A | N/A | N/A | N/A | N/A | “1”=69, “2”=97, “3”=206, “4”=78, “5”=115, “6”=46, “7”=89, “8”=61, “9”=195 | “1”=7.22, “2”=10.15, “3”=21.55, “4”=8.16, “5”=12.03, “6”=4.81, “7”=9.31, “8”=6.38, “9”=20.40 |
| V2 – round 1 | region | 956 | 0 | N/A | N/A | N/A | N/A | N/A | “1”=166, “2”=284, “3”=250, “4”=256 | “1”=17.36, “2”=29.71, “3”=26.15, “4”=26.78 |
| V2 – round 2 | region | 956 | 0 | N/A | N/A | N/A | N/A | N/A | “1”=166, “2”=284, “3”=250, “4”=256 | “1”=17.36, “2”=29.71, “3”=26.15, “4”=26.78 |
| V3 – round 1 | heatdd | 953 | 3 | 0-10816 | 4425.53 | 2199.6 | 4950 | 2460-6232 | N/A | N/A |
| V3 – round 2 | heatdd | 953 | 3 | 0-10816 | 4425.53 | 2199.6 | 4950 | 2460-6232 | N/A | N/A |
| V4 – round 1 | cooldd | 953 | 3 | 0-4389 | 1240.41 | 937.67 | 940 | 615-1566 | N/A | N/A |
| V4 – round 2 | cooldd | 953 | 3 | 0-4389 | 1240.41 | 937.67 | 940 | 615-1566 | N/A | N/A |
| V5 – round 1 | tempjan | 954 | 2 | 2.2-72.6 | 35.75 | 14.19 | 31.3 | 25.1-47.75 | N/A | N/A |
| V5 – round 2 | tempjan | 954 | 2 | 2.2-72.6 | 35.75 | 14.19 | 31.3 | 25.1-47.75 | N/A | N/A |
| V6 – round 1 | tempjuly | 954 | 2 | 58.1-93.6 | 75.05 | 5.50 | 74.25 | 71.8-78.7 | N/A | N/A |
| V6 – round 2 | tempjuly | 954 | 2 | 58.1-93.6 | 75.05 | 5.50 | 74.25 | 71.8-78.7 | N/A | N/A |
| Excel |  |  |  |  |  |  |  |  |  |  |
| V1 – round 1 | division | 956 | 0 | N/A | N/A | N/A | N/A | N/A | “1”=69, “2”=97, “3”=206, “4”=78, “5”=115, “6”=46, “7”=89, “8”=61, “9”=195 | “1”=7.22, “2”=10.15, “3”=21.55, “4”=8.16, “5”=12.03, “6”=4.81, “7”=9.31, “8”=6.38, “9”=20.40 |
| V1 – round 2 | division | 956 | 0 | N/A | N/A | N/A | N/A | N/A | “1”=69, “2”=97, “3”=206, “4”=78, “5”=115, “6”=46, “7”=89, “8”=61, “9”=195 | “1”=7.22, “2”=10.15, “3”=21.55, “4”=8.16, “5”=12.03, “6”=4.81, “7”=9.31, “8”=6.38, “9”=20.40 |
| V2 – round 1 | region | 956 | 0 | N/A | N/A | N/A | N/A | N/A | “1”=166, “2”=284, “3”=250, “4”=256 | “1”=17.36, “2”=29.71, “3”=26.15, “4”=26.78 |
| V2 – round 2 | region | 956 | 0 | N/A | N/A | N/A | N/A | N/A | “1”=166, “2”=284, “3”=250, “4”=256 | “1”=17.36, “2”=29.71, “3”=26.15, “4”=26.78 |
| V3 – round 1 | heatdd | 953 | 3 | 0-10816 | 4425.53 | 2199.6 | 4950 | 2460-6232 | N/A | N/A |
| V3 – round 2 | heatdd | 953 | 3 | 0-10816 | 4425.53 | 2199.6 | 4950 | 2460-6232 | N/A | N/A |
| V4 – round 1 | cooldd | 953 | 3 | 0-4389 | 1240.41 | 937.67 | 940 | 615-1566 | N/A | N/A |
| V4 – round 2 | cooldd | 953 | 3 | 0-4389 | 1240.41 | 937.67 | 940 | 615-1566 | N/A | N/A |
| V5 – round 1 | tempjan | 954 | 2 | 2.2-72.6 | 35.75 | 14.19 | 31.3 | 25.1-47.75 | N/A | N/A |
| V5 – round 2 | tempjan | 954 | 2 | 2.2-72.6 | 35.75 | 14.19 | 31.3 | 25.1-47.75 | N/A | N/A |
| V6 – round 1 | tempjuly | 954 | 2 | 58.1-93.6 | 75.05 | 5.50 | 74.25 | 71.8-78.7 | N/A | N/A |
| V6 – round 2 | tempjuly | 954 | 2 | 58.1-93.6 | 75.05 | 5.50 | 74.25 | 71.8-78.7 | N/A | N/A |
| Word |  |  |  |  |  |  |  |  |  |  |
| V1 – round 1 | division | 956 | 0 | N/A | N/A | N/A | N/A | N/A | “1”=69, “2”=97, “3”=206, “4”=78, “5”=115, “6”=46, “7”=89, “8”=61, “9”=195 | “1”=7.22, “2”=10.15, “3”=21.55, “4”=8.16, “5”=12.03, “6”=4.81, “7”=9.31, “8”=6.38, “9”=20.40 |
| V1 – round 2 | division | 956 | 0 | N/A | N/A | N/A | N/A | N/A | “1”=69, “2”=97, “3”=206, “4”=78, “5”=115, “6”=46, “7”=89, “8”=61, “9”=195 | “1”=7.22, “2”=10.15, “3”=21.55, “4”=8.16, “5”=12.03, “6”=4.81, “7”=9.31, “8”=6.38, “9”=20.40 |
| V2 – round 1 | region | 956 | 0 | N/A | N/A | N/A | N/A | N/A | “1”=166, “2”=284, “3”=250, “4”=256 | “1”=17.36, “2”=29.71, “3”=26.15, “4”=26.78 |
| V2 – round 2 | region | 956 | 0 | N/A | N/A | N/A | N/A | N/A | “1”=166, “2”=284, “3”=250, “4”=256 | “1”=17.36, “2”=29.71, “3”=26.15, “4”=26.78 |
| V3 – round 1 | heatdd | 953 | 3 | 0-10816 | 4425.53 | 2199.6 | 4950 | 2460-6232 | N/A | N/A |
| V3 – round 2 | heatdd | 953 | 3 | 0-10816 | 4425.53 | 2199.6 | 4950 | 2460-6232 | N/A | N/A |
| V4 – round 1 | cooldd | 953 | 3 | 0-4389 | 1240.41 | 937.67 | 940 | 615-1566 | N/A | N/A |
| V4 – round 2 | cooldd | 953 | 3 | 0-4389 | 1240.41 | 937.67 | 940 | 615-1566 | N/A | N/A |
| V5 – round 1 | tempjan | 954 | 2 | 2.2-72.6 | 35.75 | 14.19 | 31.3 | 25.1-47.75 | N/A | N/A |
| V5 – round 2 | tempjan | 954 | 2 | 2.2-72.6 | 35.75 | 14.19 | 31.3 | 25.1-47.75 | N/A | N/A |
| V6 – round 1 | tempjuly | 954 | 2 | 58.1-93.6 | 75.05 | 5.50 | 74.25 | 71.8-78.7 | N/A | N/A |
| V6 – round 2 | tempjuly | 954 | 2 | 58.1-93.6 | 75.05 | 5.50 | 74.25 | 71.8-78.7 | N/A | N/A |

**Supplementary Material 4. Claude-generated descriptive statistics for the ‘auto.dta’ and ‘citytemp.dta’ datasets across input formats and repeated runs**

| Variable | Original variable name | Non-missing observations, n | Missing observations, n | Min-Max | Mean | SD | Median | Q1-Q3 | Category frequencies, n | Category frequencies, % |
| --- | --- | --- | --- | --- | --- | --- | --- | --- | --- | --- |
| auto.dta |  |  |  |  |  |  |  |  |  |  |
| Copy paste |  |  |  |  |  |  |  |  |  |  |
| V1 – round 1 | make | N/A | N/A | N/A | N/A | N/A | N/A | N/A | N/A | N/A |
| V1 – round 2 | make | N/A | N/A | N/A | N/A | N/A | N/A | N/A | N/A | N/A |
| V2 – round 1 | price | 74 | 0 | 3291-15906 | 6165.26 | 2949.5 | 5006.5 | 4220.25-6332.25 | N/A | N/A |
| V2 – round 2 | price | 74 | 0 | 3291-15906 | 6165.26 | 2949.5 | 5006.5 | 4220.25-6332.25 | N/A | N/A |
| V3 – round 1 | mpg | 74 | 0 | 12-41 | 21.30 | 5.79 | 20 | 18-24.75 | NA | NA |
| V3 – round 2 | mpg | 74 | 0 | 12-41 | 21.30 | 5.79 | 20 | 18-24.75 | NA | NA |
| V4 – round 1 | rep78 | 69 | 5 | NA | NA | NA | NA | NA | “1”=2, “2”=8, “3”=30, “4”=18, “5”=11 | “1”=2.90, “2”=11.59, “3”=43.48, “4”=26.09, “5”=15.94 |
| V4 – round 2 | rep78 | 69 | 5 | NA | NA | NA | NA | NA | “1”=2, “2”=8, “3”=30, “4”=18, “5”=11 | “1”=2.90, “2”=11.59, “3”=43.48, “4”=26.09, “5”=15.94 |
| V5 – round 1 | headroom | 74 | 0 | NA | NA | NA | NA | NA | “1.5”=4, “2”=13, “2.5”=14, “3”=13, “3.5”=15, “4”=10, “4.5”=4, “5”=1 | “1.5”=5.41, “2”=17.57, “2.5”=18.92, “3”=17.57, “3.5”=20.27, “4”=13.51, “4.5”=5.41, “5”=1.35 |
| V5 – round 2 | headroom | 74 | 0 | NA | NA | NA | NA | NA | “1.5”=4, “2”=13, “2.5”=14, “3”=13, “3.5”=15, “4”=10, “4.5”=4, “5”=1 | “1.5”=5.41, “2”=17.57, “2.5”=18.92, “3”=17.57, “3.5”=20.27, “4”=13.51, “4.5”=5.41, “5”=1.35 |
| V6 – round 1 | trunk | 74 | 0 | 5-23 | 13.76 | 4.28 | 14 | 10.25-16.75 | N/A | N/A |
| V6 – round 2 | trunk | 74 | 0 | 5-23 | 13.76 | 4.28 | 14 | 10.25-16.75 | N/A | N/A |
| V7 – round 1 | weight | 74 | 0 | 1760-4840 | 3019.46 | 777.19 | 3190 | 2250-3600 | N/A | N/A |
| V7 – round 2 | weight | 74 | 0 | 1760-4840 | 3019.46 | 777.19 | 3190 | 2250-3600 | N/A | N/A |
| V8 – round 1 | length | 74 | 0 | 142-233 | 187.93 | 22.27 | 192.5 | 170-203.75 | N/A | N/A |
| V8 – round 2 | length | 74 | 0 | 142-233 | 187.93 | 22.27 | 192.5 | 170-203.75 | N/A | N/A |
| V9 – round 1 | turn | 74 | 0 | 31-51 | 39.65 | 4.40 | 40 | 36-43 | N/A | N/A |
| V9 – round 2 | turn | 74 | 0 | 31-51 | 39.65 | 4.40 | 40 | 36-43 | N/A | N/A |
| V10 – round 1 | displacement | 74 | 0 | 79-425 | 197.30 | 91.84 | 196 | 119-245.25 | N/A | N/A |
| V10 – round 2 | displacement | 74 | 0 | 79-425 | 197.30 | 91.84 | 196 | 119-245.25 | N/A | N/A |
| V11 – round 1 | gear_ratio | 74 | 0 | 2.19-3.89 | 3.01 | 0.46 | 2.96 | 2.73-3.35 | N/A | N/A |
| V11 – round 2 | gear_ratio | 74 | 0 | 2.19-3.89 | 3.01 | 0.46 | 2.96 | 2.73-3.35 | N/A | N/A |
| V12 – round 1 | foreign | 74 | 0 | N/A | N/A | N/A | N/A | N/A | “0”=52, “1”=22 | “0”=70.27, “1”=29.73 |
| V12 – round 2 | foreign | 74 | 0 | N/A | N/A | N/A | N/A | N/A | “0”=52, “1”=22 | “0”=70.27, “1”=29.73 |
| Excel |  |  |  |  |  |  |  |  |  |  |
| V1 – round 1 | make | N/A | N/A | N/A | N/A | N/A | N/A | N/A | N/A | N/A |
| V1 – round 2 | make | N/A | N/A | N/A | N/A | N/A | N/A | N/A | N/A | N/A |
| V2 – round 1 | price | 74 | 0 | 3291-15906 | 6165.26 | 2949.5 | 5006.5 | 4220.25-6332.25 | N/A | N/A |
| V2 – round 2 | price | 74 | 0 | 3291-15906 | 6165.26 | 2949.5 | 5006.5 | 4220.25-6332.25 | N/A | N/A |
| V3 – round 1 | mpg | 74 | 0 | 12-41 | 21.30 | 5.79 | 20 | 18-24.75 | NA | NA |
| V3 – round 2 | mpg | 74 | 0 | 12-41 | 21.30 | 5.79 | 20 | 18-24.75 | NA | NA |
| V4 – round 1 | rep78 | 69 | 5 | NA | NA | NA | NA | NA | “1”=2, “2”=8, “3”=30, “4”=18, “5”=11 | “1”=2.90, “2”=11.59, “3”=43.48, “4”=26.09, “5”=15.94 |
| V4 – round 2 | rep78 | 69 | 5 | NA | NA | NA | NA | NA | “1”=2, “2”=8, “3”=30, “4”=18, “5”=11 | “1”=2.90, “2”=11.59, “3”=43.48, “4”=26.09, “5”=15.94 |
| V5 – round 1 | headroom | 74 | 0 | NA | NA | NA | NA | NA | “1.5”=4, “2”=13, “2.5”=14, “3”=13, “3.5”=15, “4”=10, “4.5”=4, “5”=1 | “1.5”=5.41, “2”=17.57, “2.5”=18.92, “3”=17.57, “3.5”=20.27, “4”=13.51, “4.5”=5.41, “5”=1.35 |
| V5 – round 2 | headroom | 74 | 0 | NA | NA | NA | NA | NA | “1.5”=4, “2”=13, “2.5”=14, “3”=13, “3.5”=15, “4”=10, “4.5”=4, “5”=1 | “1.5”=5.41, “2”=17.57, “2.5”=18.92, “3”=17.57, “3.5”=20.27, “4”=13.51, “4.5”=5.41, “5”=1.35 |
| V6 – round 1 | trunk | 74 | 0 | 5-23 | 13.76 | 4.28 | 14 | 10.25-16.75 | N/A | N/A |
| V6 – round 2 | trunk | 74 | 0 | 5-23 | 13.76 | 4.28 | 14 | 10.25-16.75 | N/A | N/A |
| V7 – round 1 | weight | 74 | 0 | 1760-4840 | 3019.46 | 777.19 | 3190 | 2250-3600 | N/A | N/A |
| V7 – round 2 | weight | 74 | 0 | 1760-4840 | 3019.46 | 777.19 | 3190 | 2250-3600 | N/A | N/A |
| V8 – round 1 | length | 74 | 0 | 142-233 | 187.93 | 22.27 | 192.5 | 170-203.75 | N/A | N/A |
| V8 – round 2 | length | 74 | 0 | 142-233 | 187.93 | 22.27 | 192.5 | 170-203.75 | N/A | N/A |
| V9 – round 1 | turn | 74 | 0 | 31-51 | 39.65 | 4.40 | 40 | 36-43 | N/A | N/A |
| V9 – round 2 | turn | 74 | 0 | 31-51 | 39.65 | 4.40 | 40 | 36-43 | N/A | N/A |
| V10 – round 1 | displacement | 74 | 0 | 79-425 | 197.30 | 91.84 | 196 | 119-245.25 | N/A | N/A |
| V10 – round 2 | displacement | 74 | 0 | 79-425 | 197.30 | 91.84 | 196 | 119-245.25 | N/A | N/A |
| V11 – round 1 | gear_ratio | 74 | 0 | 2.19-3.89 | 3.01 | 0.46 | 2.96 | 2.73-3.35 | N/A | N/A |
| V11 – round 2 | gear_ratio | 74 | 0 | 2.19-3.89 | 3.01 | 0.46 | 2.96 | 2.73-3.35 | N/A | N/A |
| V12 – round 1 | foreign | 74 | 0 | N/A | N/A | N/A | N/A | N/A | “0”=52, “1”=22 | “0”=70.27, “1”=29.73 |
| V12 – round 2 | foreign | 74 | 0 | N/A | N/A | N/A | N/A | N/A | “0”=52, “1”=22 | “0”=70.27, “1”=29.73 |
| Word |  |  |  |  |  |  |  |  |  |  |
| V1 – round 1 | make | N/A | N/A | N/A | N/A | N/A | N/A | N/A | N/A | N/A |
| V1 – round 2 | make | N/A | N/A | N/A | N/A | N/A | N/A | N/A | N/A | N/A |
| V2 – round 1 | price | 74 | 0 | 3291-15906 | 6165.26 | 2949.5 | 5006.5 | 4220.25-6332.25 | N/A | N/A |
| V2 – round 2 | price | 74 | 0 | 3291-15906 | 6165.26 | 2949.5 | 5006.5 | 4220.25-6332.25 | N/A | N/A |
| V3 – round 1 | mpg | 74 | 0 | 12-41 | 21.30 | 5.79 | 20 | 18-24.75 | NA | NA |
| V3 – round 2 | mpg | 74 | 0 | 12-41 | 21.30 | 5.79 | 20 | 18-24.75 | NA | NA |
| V4 – round 1 | rep78 | 69 | 5 | NA | NA | NA | NA | NA | “1”=2, “2”=8, “3”=30, “4”=18, “5”=11 | “1”=2.90, “2”=11.59, “3”=43.48, “4”=26.09, “5”=15.94 |
| V4 – round 2 | rep78 | 69 | 5 | NA | NA | NA | NA | NA | “1”=2, “2”=8, “3”=30, “4”=18, “5”=11 | “1”=2.90, “2”=11.59, “3”=43.48, “4”=26.09, “5”=15.94 |
| V5 – round 1 | headroom | 74 | 0 | NA | NA | NA | NA | NA | “1.5”=4, “2”=13, “2.5”=14, “3”=13, “3.5”=15, “4”=10, “4.5”=4, “5”=1 | “1.5”=5.41, “2”=17.57, “2.5”=18.92, “3”=17.57, “3.5”=20.27, “4”=13.51, “4.5”=5.41, “5”=1.35 |
| V5 – round 2 | headroom | 74 | 0 | NA | NA | NA | NA | NA | “1.5”=4, “2”=13, “2.5”=14, “3”=13, “3.5”=15, “4”=10, “4.5”=4, “5”=1 | “1.5”=5.41, “2”=17.57, “2.5”=18.92, “3”=17.57, “3.5”=20.27, “4”=13.51, “4.5”=5.41, “5”=1.35 |
| V6 – round 1 | trunk | 74 | 0 | 5-23 | 13.76 | 4.28 | 14 | 10.25-16.75 | N/A | N/A |
| V6 – round 2 | trunk | 74 | 0 | 5-23 | 13.76 | 4.28 | 14 | 10.25-16.75 | N/A | N/A |
| V7 – round 1 | weight | 74 | 0 | 1760-4840 | 3019.46 | 777.19 | 3190 | 2250-3600 | N/A | N/A |
| V7 – round 2 | weight | 74 | 0 | 1760-4840 | 3019.46 | 777.19 | 3190 | 2250-3600 | N/A | N/A |
| V8 – round 1 | length | 74 | 0 | 142-233 | 187.93 | 22.27 | 192.5 | 170-203.75 | N/A | N/A |
| V8 – round 2 | length | 74 | 0 | 142-233 | 187.93 | 22.27 | 192.5 | 170-203.75 | N/A | N/A |
| V9 – round 1 | turn | 74 | 0 | 31-51 | 39.65 | 4.40 | 40 | 36-43 | N/A | N/A |
| V9 – round 2 | turn | 74 | 0 | 31-51 | 39.65 | 4.40 | 40 | 36-43 | N/A | N/A |
| V10 – round 1 | displacement | 74 | 0 | 79-425 | 197.30 | 91.84 | 196 | 119-245.25 | N/A | N/A |
| V10 – round 2 | displacement | 74 | 0 | 79-425 | 197.30 | 91.84 | 196 | 119-245.25 | N/A | N/A |
| V11 – round 1 | gear_ratio | 74 | 0 | 2.19-3.89 | 3.01 | 0.46 | 2.96 | 2.73-3.35 | N/A | N/A |
| V11 – round 2 | gear_ratio | 74 | 0 | 2.19-3.89 | 3.01 | 0.46 | 2.96 | 2.73-3.35 | N/A | N/A |
| V12 – round 1 | foreign | 74 | 0 | N/A | N/A | N/A | N/A | N/A | “0”=52, “1”=22 | “0”=70.27, “1”=29.73 |
| V12 – round 2 | foreign | 74 | 0 | N/A | N/A | N/A | N/A | N/A | “0”=52, “1”=22 | “0”=70.27, “1”=29.73 |
| citytemp.dta |  |  |  |  |  |  |  |  |  |  |
| Copy paste |  |  |  |  |  |  |  |  |  |  |
| V1 – round 1 | division | 956 | 0 | N/A | N/A | N/A | N/A | N/A | “1”=69, “2”=97, “3”=206, “4”=78, “5”=115, “6”=46, “7”=89, “8”=61, “9”=195 | “1”=7.22, “2”=10.15, “3”=21.55, “4”=8.16, “5”=12.03, “6”=4.81, “7”=9.31, “8”=6.38, “9”=20.40 |
| V1 – round 2 | division | 956 | 0 | N/A | N/A | N/A | N/A | N/A | “1”=69, “2”=97, “3”=206, “4”=78, “5”=115, “6”=46, “7”=89, “8”=61, “9”=195 | “1”=7.22, “2”=10.15, “3”=21.55, “4”=8.16, “5”=12.03, “6”=4.81, “7”=9.31, “8”=6.38, “9”=20.40 |
| V2 – round 1 | region | 956 | 0 | N/A | N/A | N/A | N/A | N/A | “1”=166, “2”=284, “3”=250, “4”=256 | “1”=17.36, “2”=29.71, “3”=26.15, “4”=26.78 |
| V2 – round 2 | region | 956 | 0 | N/A | N/A | N/A | N/A | N/A | “1”=166, “2”=284, “3”=250, “4”=256 | “1”=17.36, “2”=29.71, “3”=26.15, “4”=26.78 |
| V3 – round 1 | heatdd | 953 | 3 | 0-10816 | 4425.53 | 2199.6 | 4950 | 2460-6232 | N/A | N/A |
| V3 – round 2 | heatdd | 953 | 3 | 0-10816 | 4425.53 | 2199.6 | 4950 | 2460-6232 | N/A | N/A |
| V4 – round 1 | cooldd | 953 | 3 | 0-4389 | 1240.41 | 937.67 | 940 | 615-1566 | N/A | N/A |
| V4 – round 2 | cooldd | 953 | 3 | 0-4389 | 1240.41 | 937.67 | 940 | 615-1566 | N/A | N/A |
| V5 – round 1 | tempjan | 954 | 2 | 2.2-72.6 | 35.75 | 14.19 | 31.3 | 25.1-47.75 | N/A | N/A |
| V5 – round 2 | tempjan | 954 | 2 | 2.2-72.6 | 35.75 | 14.19 | 31.3 | 25.1-47.75 | N/A | N/A |
| V6 – round 1 | tempjuly | 954 | 2 | 58.1-93.6 | 75.05 | 5.50 | 74.25 | 71.8-78.7 | N/A | N/A |
| V6 – round 2 | tempjuly | 954 | 2 | 58.1-93.6 | 75.05 | 5.50 | 74.25 | 71.8-78.7 | N/A | N/A |
| Excel |  |  |  |  |  |  |  |  |  |  |
| V1 – round 1 | division | 956 | 0 | N/A | N/A | N/A | N/A | N/A | “1”=69, “2”=97, “3”=206, “4”=78, “5”=115, “6”=46, “7”=89, “8”=61, “9”=195 | “1”=7.22, “2”=10.15, “3”=21.55, “4”=8.16, “5”=12.03, “6”=4.81, “7”=9.31, “8”=6.38, “9”=20.40 |
| V1 – round 2 | division | 956 | 0 | N/A | N/A | N/A | N/A | N/A | “1”=69, “2”=97, “3”=206, “4”=78, “5”=115, “6”=46, “7”=89, “8”=61, “9”=195 | “1”=7.22, “2”=10.15, “3”=21.55, “4”=8.16, “5”=12.03, “6”=4.81, “7”=9.31, “8”=6.38, “9”=20.40 |
| V2 – round 1 | region | 956 | 0 | N/A | N/A | N/A | N/A | N/A | “1”=166, “2”=284, “3”=250, “4”=256 | “1”=17.36, “2”=29.71, “3”=26.15, “4”=26.78 |
| V2 – round 2 | region | 956 | 0 | N/A | N/A | N/A | N/A | N/A | “1”=166, “2”=284, “3”=250, “4”=256 | “1”=17.36, “2”=29.71, “3”=26.15, “4”=26.78 |
| V3 – round 1 | heatdd | 953 | 3 | 0-10816 | 4425.53 | 2199.6 | 4950 | 2460-6232 | N/A | N/A |
| V3 – round 2 | heatdd | 953 | 3 | 0-10816 | 4425.53 | 2199.6 | 4950 | 2460-6232 | N/A | N/A |
| V4 – round 1 | cooldd | 953 | 3 | 0-4389 | 1240.41 | 937.67 | 940 | 615-1566 | N/A | N/A |
| V4 – round 2 | cooldd | 953 | 3 | 0-4389 | 1240.41 | 937.67 | 940 | 615-1566 | N/A | N/A |
| V5 – round 1 | tempjan | 954 | 2 | 2.2-72.6 | 35.75 | 14.19 | 31.3 | 25.1-47.75 | N/A | N/A |
| V5 – round 2 | tempjan | 954 | 2 | 2.2-72.6 | 35.75 | 14.19 | 31.3 | 25.1-47.75 | N/A | N/A |
| V6 – round 1 | tempjuly | 954 | 2 | 58.1-93.6 | 75.05 | 5.50 | 74.25 | 71.8-78.7 | N/A | N/A |
| V6 – round 2 | tempjuly | 954 | 2 | 58.1-93.6 | 75.05 | 5.50 | 74.25 | 71.8-78.7 | N/A | N/A |
| Word |  |  |  |  |  |  |  |  |  |  |
| V1 – round 1 | division | 956 | 0 | N/A | N/A | N/A | N/A | N/A | “1”=69, “2”=97, “3”=206, “4”=78, “5”=115, “6”=46, “7”=89, “8”=61, “9”=195 | “1”=7.22, “2”=10.15, “3”=21.55, “4”=8.16, “5”=12.03, “6”=4.81, “7”=9.31, “8”=6.38, “9”=20.40 |
| V1 – round 2 | division | 956 | 0 | N/A | N/A | N/A | N/A | N/A | “1”=69, “2”=97, “3”=206, “4”=78, “5”=115, “6”=46, “7”=89, “8”=61, “9”=195 | “1”=7.22, “2”=10.15, “3”=21.55, “4”=8.16, “5”=12.03, “6”=4.81, “7”=9.31, “8”=6.38, “9”=20.40 |
| V2 – round 1 | region | 956 | 0 | N/A | N/A | N/A | N/A | N/A | “1”=166, “2”=284, “3”=250, “4”=256 | “1”=17.36, “2”=29.71, “3”=26.15, “4”=26.78 |
| V2 – round 2 | region | 956 | 0 | N/A | N/A | N/A | N/A | N/A | “1”=166, “2”=284, “3”=250, “4”=256 | “1”=17.36, “2”=29.71, “3”=26.15, “4”=26.78 |
| V3 – round 1 | heatdd | 953 | 3 | 0-10816 | 4425.53 | 2199.6 | 4950 | 2460-6232 | N/A | N/A |
| V3 – round 2 | heatdd | 953 | 3 | 0-10816 | 4425.53 | 2199.6 | 4950 | 2460-6232 | N/A | N/A |
| V4 – round 1 | cooldd | 953 | 3 | 0-4389 | 1240.41 | 937.67 | 940 | 615-1566 | N/A | N/A |
| V4 – round 2 | cooldd | 953 | 3 | 0-4389 | 1240.41 | 937.67 | 940 | 615-1566 | N/A | N/A |
| V5 – round 1 | tempjan | 954 | 2 | 2.2-72.6 | 35.75 | 14.19 | 31.3 | 25.1-47.75 | N/A | N/A |
| V5 – round 2 | tempjan | 954 | 2 | 2.2-72.6 | 35.75 | 14.19 | 31.3 | 25.1-47.75 | N/A | N/A |
| V6 – round 1 | tempjuly | 954 | 2 | 58.1-93.6 | 75.05 | 5.50 | 74.25 | 71.8-78.7 | N/A | N/A |
| V6 – round 2 | tempjuly | 954 | 2 | 58.1-93.6 | 75.05 | 5.50 | 74.25 | 71.8-78.7 | N/A | N/A |

**Supplementary Material 5. Reference descriptive statistics obtained with Stata**

**auto.dta**

Contains data	1978 Automobile Data

obs: 74	13 Apr 2016 17:45

vars: 12

size: 3,478

-------------------------------------------------------------------------------

storage	display	value

variable name	type	format	label	variable label

----------------------------------------------------------------------------------------------------------------------------------------------------------------

make	str18	%-18s	Make and Model

price	int	%8.0gc	Price

mpg	int	%8.0g	Mileage (mpg)

rep78	int	%8.0g	Repair Record 1978

headroom	float	%6.1f	Headroom (in.)

trunk	int	%8.0g	Trunk space (cu. ft.)

weight	int	%8.0gc	Weight (lbs.)

length	int	%8.0g	Length (in.)

turn	int	%8.0g	Turn Circle (ft.)

displacement	int	%8.0g	Displacement (cu. in.)

gear_ratio	float	%6.2f	Gear Ratio

foreign	byte	%8.0g	origin	Car type

-------------------------------------------------------------------------------

----------------------------------------------------------------------------------------------------------------------------------------------------------------

make	Make and Model

----------------------------------------------------------------------------------------------------------------------------------------------------------------

type: string (str18), but longest is str17

unique values: 74	missing "": 0/74

examples: "Cad. Deville"

"Dodge Magnum"

"Merc. XR-7"

"Pont. Catalina"

warning: variable has embedded blanks

----------------------------------------------------------------------------------------------------------------------------------------------------------------

price	Price

----------------------------------------------------------------------------------------------------------------------------------------------------------------

type: numeric (int)

range: [3291,15906]	units: 1

unique values: 74	missing .: 0/74

mean: 6165.26

std. dev: 2949.5

percentiles: 10%	25%	50%	75%	90%

3895	4195	5006.5	6342	11385

----------------------------------------------------------------------------------------------------------------------------------------------------------------

mpg	Mileage (mpg)

----------------------------------------------------------------------------------------------------------------------------------------------------------------

type: numeric (int)

range: [12,41]	units: 1

unique values: 21	missing .: 0/74

mean: 21.2973

std. dev: 5.7855

percentiles: 10%	25%	50%	75%	90%

14	18	20	25	29

----------------------------------------------------------------------------------------------------------------------------------------------------------------

rep78	Repair Record 1978

----------------------------------------------------------------------------------------------------------------------------------------------------------------

type: numeric (int)

range: [1,5]	units: 1

unique values: 5	missing .: 5/74

tabulation: Freq. Value

2 1

8 2

30 3

18 4

11 5

5 .

----------------------------------------------------------------------------------------------------------------------------------------------------------------

headroom	Headroom (in.)

----------------------------------------------------------------------------------------------------------------------------------------------------------------

type: numeric (float)

range: [1.5,5]	units: .1

unique values: 8	missing .: 0/74

tabulation: Freq. Value

4 1.5

13 2

14 2.5

13 3

15 3.5

10 4

4 4.5

1 5

----------------------------------------------------------------------------------------------------------------------------------------------------------------

trunk	Trunk space (cu. ft.)

----------------------------------------------------------------------------------------------------------------------------------------------------------------

type: numeric (int)

range: [5,23]	units: 1

unique values: 18	missing .: 0/74

mean: 13.7568

std. dev: 4.2774

percentiles: 10%	25%	50%	75%	90%

8	10	14	17	20

----------------------------------------------------------------------------------------------------------------------------------------------------------------

weight	Weight (lbs.)

----------------------------------------------------------------------------------------------------------------------------------------------------------------

type: numeric (int)

range: [1760,4840]	units: 10

unique values: 64	missing .: 0/74

mean: 3019.46

std. dev: 777.194

percentiles: 10%	25%	50%	75%	90%

2020	2240	3190	3600	4060

----------------------------------------------------------------------------------------------------------------------------------------------------------------

length	Length (in.)

----------------------------------------------------------------------------------------------------------------------------------------------------------------

type: numeric (int)

range: [142,233]	units: 1

unique values: 47	missing .: 0/74

mean: 187.932

std. dev: 22.2663

percentiles: 10%	25%	50%	75%	90%

157	170	192.5	204	218

----------------------------------------------------------------------------------------------------------------------------------------------------------------

turn	Turn Circle (ft.)

----------------------------------------------------------------------------------------------------------------------------------------------------------------

type: numeric (int)

range: [31,51]	units: 1

unique values: 18	missing .: 0/74

mean: 39.6486

std. dev: 4.39935

percentiles: 10%	25%	50%	75%	90%

34	36	40	43	45

----------------------------------------------------------------------------------------------------------------------------------------------------------------

displacement	Displacement (cu. in.)

----------------------------------------------------------------------------------------------------------------------------------------------------------------

type: numeric (int)

range: [79,425]	units: 1

unique values: 31	missing .: 0/74

mean: 197.297

std. dev: 91.8372

percentiles: 10%	25%	50%	75%	90%

97	119	196	250	350

----------------------------------------------------------------------------------------------------------------------------------------------------------------

gear_ratio	Gear Ratio

----------------------------------------------------------------------------------------------------------------------------------------------------------------

type: numeric (float)

range: [2.19,3.89]	units: .01

unique values: 36	missing .: 0/74

mean: 3.01486

std. dev: .456287

percentiles: 10%	25%	50%	75%	90%

2.43	2.73	2.955	3.37	3.72

----------------------------------------------------------------------------------------------------------------------------------------------------------------

foreign	Car type

----------------------------------------------------------------------------------------------------------------------------------------------------------------

type: numeric (byte)

label: origin

range: [0,1]	units: 1

unique values: 2	missing .: 0/74

tabulation: Freq. Numeric Label

52	0 Domestic

22	1 Foreign

**citytemp.dta**

Contains data	City Temperature Data

obs: 956	3 Mar 2016 19:17

vars: 6

size: 19,120

-------------------------------------------------------------------------------

storage	display	value

variable name	type	format	label	variable label

--------------------------------------------------------------------------------------------------------------------------------------------------------------------------------------------------------

division	int	%8.0g	division	Census Division

region	int	%8.0g	region	Census Region

heatdd	int	%8.0g	Heating degree days

cooldd	int	%8.0g	Cooling degree days

tempjan	float	%9.0g	Average January temperature

tempjuly	float	%9.0g	Average July temperature

-------------------------------------------------------------------------------

--------------------------------------------------------------------------------------------------------------------------------------------------------------------------------------------------------

division	Census Division

--------------------------------------------------------------------------------------------------------------------------------------------------------------------------------------------------------

type: numeric (int)

label: division

range: [1,9]	units: 1

unique values: 9	missing .: 0/956

tabulation: Freq. Numeric Label

69	1 N. Eng.

97	2 Mid Atl

206	3 E.N.C.

78	4 W.N.C.

115	5 S. Atl.

46	6 E.S.C.

89	7 W.S.C.

61	8 Mountain

195	9 Pacific

--------------------------------------------------------------------------------------------------------------------------------------------------------------------------------------------------------

region	Census Region

--------------------------------------------------------------------------------------------------------------------------------------------------------------------------------------------------------

type: numeric (int)

label: region

range: [1,4]	units: 1

unique values: 4	missing .: 0/956

tabulation: Freq. Numeric Label

166	1 NE

284	2 N Cntrl

250	3 South

256	4 West

--------------------------------------------------------------------------------------------------------------------------------------------------------------------------------------------------------

heatdd	Heating degree days

--------------------------------------------------------------------------------------------------------------------------------------------------------------------------------------------------------

type: numeric (int)

range: [0,10816]	units: 1

unique values: 471	missing .: 3/956

mean: 4425.53

std. dev: 2199.6

percentiles: 10%	25%	50%	75%	90%

1510	2460	4950	6232	6919

--------------------------------------------------------------------------------------------------------------------------------------------------------------------------------------------------------

cooldd	Cooling degree days

--------------------------------------------------------------------------------------------------------------------------------------------------------------------------------------------------------

type: numeric (int)

range: [0,4389]	units: 1

unique values: 438	missing .: 3/956

mean: 1240.41

std. dev: 937.668

percentiles: 10%	25%	50%	75%	90%

411	615	940	1566	2761

--------------------------------------------------------------------------------------------------------------------------------------------------------------------------------------------------------

tempjan	Average January temperature

--------------------------------------------------------------------------------------------------------------------------------------------------------------------------------------------------------

type: numeric (float)

range: [2.2,72.6]	units: .1

unique values: 310	missing .: 2/956

mean: 35.749

std. dev: 14.1881

percentiles: 10%	25%	50%	75%	90%

20.2	25.1	31.3	47.8	55.1

--------------------------------------------------------------------------------------------------------------------------------------------------------------------------------------------------------

tempjuly	Average July temperature

--------------------------------------------------------------------------------------------------------------------------------------------------------------------------------------------------------

type: numeric (float)

range: [58.1,93.6]	units: .1

unique values: 196	missing .: 2/956

mean: 75.0538

std. dev: 5.49504

percentiles: 10%	25%	50%	75%	90%

68.8	71.8	74.25	78.7	82.3
